# Anion-enrichment interface enables high-voltage anode-free lithium metal batteries

**DOI:** 10.1038/s41467-023-36853-x

**Published:** 2023-02-25

**Authors:** Minglei Mao, Xiao Ji, Qiyu Wang, Zejing Lin, Meiying Li, Tao Liu, Chengliang Wang, Yong-Sheng Hu, Hong Li, Xuejie Huang, Liquan Chen, Liumin Suo

**Affiliations:** 1 Beijing Advanced Innovation Center for Materials Genome Engineering, Institute of Physics, Chinese Academy of Sciences, Beijing National Laboratory for Condensed Matter Physics, Beijing, 100190 China; 2 School of Integrated Circuits, School of Optical and Electronic Information, Wuhan National Laboratory for Optoelectronics (WNLO), Optics Valley Laboratory, Huazhong University of Science and Technology, Wuhan, 430074 China

**Keywords:** Batteries, Batteries, Batteries

## Abstract

Aggressive chemistry involving Li metal anode (LMA) and high-voltage LiNi_0.8_Mn_0.1_Co_0.1_O_2_ (NCM811) cathode is deemed as a pragmatic approach to pursue the desperate 400 Wh kg^−1^. Yet, their implementation is plagued by low Coulombic efficiency and inferior cycling stability. Herein, we propose an optimally fluorinated linear carboxylic ester (ethyl 3,3,3-trifluoropropanoate, FEP) paired with weakly solvating fluoroethylene carbonate and dissociated lithium salts (LiBF_4_ and LiDFOB) to prepare a weakly solvating and dissociated electrolyte. An anion-enrichment interface prompts more anions’ decomposition in the inner Helmholtz plane and higher reduction potential of anions. Consequently, the anion-derived interface chemistry contributes to the compact and columnar-structure Li deposits with a high CE of 98.7% and stable cycling of 4.6 V NCM811 and LiCoO_2_ cathode. Accordingly, industrial anode-free pouch cells under harsh testing conditions deliver a high energy of 442.5 Wh kg^−1^ with 80% capacity retention after 100 cycles.

## Abstract (web summary)

The implementation of Li metal anode with high-voltage Ni/Co rich cathode is plagued by low coulombic efficiency and inferior cycling stability. Here authors propose an anion-enriched interface to facilitate the columnar-structure of Li deposits to solve this issue.

## Introduction

Lithium-ion batteries (LIBs) are witnessing a huge surge in demand for portable electronics, electric vehicles, and grid storage applications. Yet, current LIBs chemistry, pairing lithium transition metal oxide cathodes (LiCoO_2_, LiMn_2_O_4_, LiFePO_4_, etc.)^1^ with a graphite anode (theoretical specific capacity of 372 mAh g^−1^)^2^, is approaching the ceilings of energy density (~300 Wh kg^−1^)^3^ and struggling to satisfy the ballooning demands. To pursue the desperate 400 Wh kg^−1^ at the cell level, a practicable alternative is to resort to more aggressive chemistries combining LMA with the high-voltage and high-capacity Ni-rich NCM (LiNi_x_M_1-x_O_2_, M = Mn, Co, and *x* > 0.6) cathode^4–6^.

Lithium metal is the ultimate anode choice for high-energy battery systems due to its low potential (−3.04 V vs. SHE) and high specific capacity (3860 mAh g^−1^). However, the implementation of LMA is afflicted by its poor cycle stability and safety issues^7^. Uncontrollable side reactions between Li metal and electrolytes will form a chemically unstable and mechanically fragile solid-electrolyte interphase (SEI)^8^. The SEI readily cracks over repeated Li deposition and stripping, leading to dendritic growth^9^, formation of dead Li^10^, and continuous consumption of Li inventory^11,12^. Worse still, the constant fracture and reconstruction of SEI will cause electrolytes rapidly depleted or contaminated by side reactions^4^. Loss of ionic percolation resultantly arises due to the wetting of large-surface-area and large-thickness Li deposits. Artificial SEI^13^, 3D current collectors^14^, and separator modification^15^ have been applied to respond to the challenge and achieved some success. Recently, battery communities come to realize that the behavior of Li metal is primarily determined by its interfacial chemistry which is closely related to electrolyte engineering.

For high-nickel NCM cathodes, especially LiNi_0.8_Co_0.1_Mn_0.1_O_2_ (NCM811) of commercial interest, simply extending the upper cut-off voltage is an effective means to boost the capacity and energy density^16^, which unfortunately will intensify cathode degradation reactions^17,18^. Several mechanisms have been proposed to account for the performance deterioration of NCM cathode, including irreversible phase transition^19,20^, gas generation^21^, transition metal (TM) dissolution^22^, stress-corrosion cracking of the NCM secondary particles^23,24^, and unceasing growth of cathode-electrolyte interphases (CEIs)^20^. The thermodynamically unstable delithiated NCM811 is subject to phase transition from layered phase over disordered spinel phase to the NiO-like rock-salt phase, accompanied by capacity loss and oxygen evolution. Phase transition and oxygen evolution induce the formation of microcracks in NCM secondary particles. The electrolyte will penetrate into the microcracks and react with the delithiated cathodes, which therein forms a resistive surface layer and elevates the cell’s impedance^25,26^. Furthermore, dissolved TMs will migrate to the anode side and be deposited, resulting in the destruction of the SEI and impedance growth^27^. These degradation mechanisms of NCM cathode are usually initiated from its interface^28,29^, which renders the on-site interface modification by electrolyte engineering extremely important for applying high-Ni cathode.

As discussed above, electrolyte engineering will modify the interface chemistry of both LMA and NCM811 cathode and thus is deemed as a critical and practicable alternative to implement the high-voltage lithium-metal batteries (LMBs). It is well established that the classic electrode-electrolyte interphases (EEIs), including SEIs and CEIs, are primarily solvent-derived and consist of a majority of organic and some inorganic compounds^30–32^. The incompact and unsolidified mosaic-type EEIs hardly support long-cycling LMA and high-voltage NCM811 cathodes. In contrast, anion-derived EEIs are mainly composed of inorganic lithium compounds (such as LiF, Li_2_O, etc.)^33–35^, namely inorganic-abundant EEIs. For LMA, the inorganic-abundant SEIs with a high Young’s modulus are mechanically strong to restrain the Li dendritic growth and penetration into the interface^36^. Besides, the inorganic-abundant SEIs with high interfacial energy weakly bond with Li, which promotes the Li lateral diffusion along the interface and suppresses metallic Li from penetrating the SEIs^37–39^. Regarding NCM811 cathode, the compact and uniform inorganic-abundant CEIs will facilitate the lithium-ion transport and besides prevent the permeation of liquid electrolyte along grain boundaries of Ni-rich cathodes, significantly mitigating the deleterious cathode-electrolyte side reaction^40,41^.

The interfacial chemistry is dictated by the solvation structure of electrolytes that depends on the competitive coordination between solvent and anion ligands with Li^+^ through ion-dipole or ion-ion interactions^42–44^. In conventional dilute electrolytes (~1.0 M), the Li^+^ solvation sheath is dominated by strongly solvating polar solvents with most anions excluded^45,46^. As the precursor of EEIs, such primary solvation sheath results in the solvent-derived interphasial chemistry^47–49^. To enable anion-derived interfacial chemistry, a routine solution is to straightforwardly increase the ratio of anions to solvents as in the concept of super-concentrated electrolyte (SCE)^50–52^. Due to the scarcity of solvents and abundance of anions, anions inevitably appear in the primary solvation sheath of Li^+^ to form contact ion pairs (CIP) or aggregates (AGG) with decoupling the interaction between Li^+^ and solvents^53,54^. Nevertheless, the implementation of SCE might be plagued by the high viscosity, poor wettability toward polyolefin separators and electrodes, and high cost^50^.

Instead of temerariously increasing salt concentration in SCE, simultaneously tailoring the intrinsic solvating power of solvents and dissociation constant of lithium salts is proposed as a more effective approach towards anion-rich solvation structure and anion-derived interfacial chemistry in the dilute electrolyte (<2.0 M). As discussed above, solvents and anions are vying to enter the solvation sheath of Li^+^. Solvents with low solvating power will be defeated by anions of weakly dissociated lithium salts^30,55^, resulting in anions prevailing to coordinate with Li^+^. The weakly solvating and dissociated electrolyte (WSDE) will yield abundant CIPs and AGGs at low salt concentrations, leading to anion-derived interfacial chemistry.

In this work, we propose an optimal fluorinated linear carboxylic ester (ethyl 3,3,3-trifluoropropanoate, tFEP) paired with weakly solvating fluoroethylene carbonate (FEC) and dissociated lithium salts (LiBF_4_ and LiDFOB) to prepare a WSDE. In fluorinated linear carboxylic esters, we introduce the –F group to precisely manipulate the solvating power and facilitate the LiF-abundant interfacial chemistry, in which tFEP has the optimal solvating power and permittivity. An anion-enrichment interface is formed in the WSDE electrolyte, contributing to the stable cycling of LMA and aggressive NCM811 and LiCoO_2_ cathode.

## Results

### Design principle of anion-enrichment interface

**Fig. 1:**
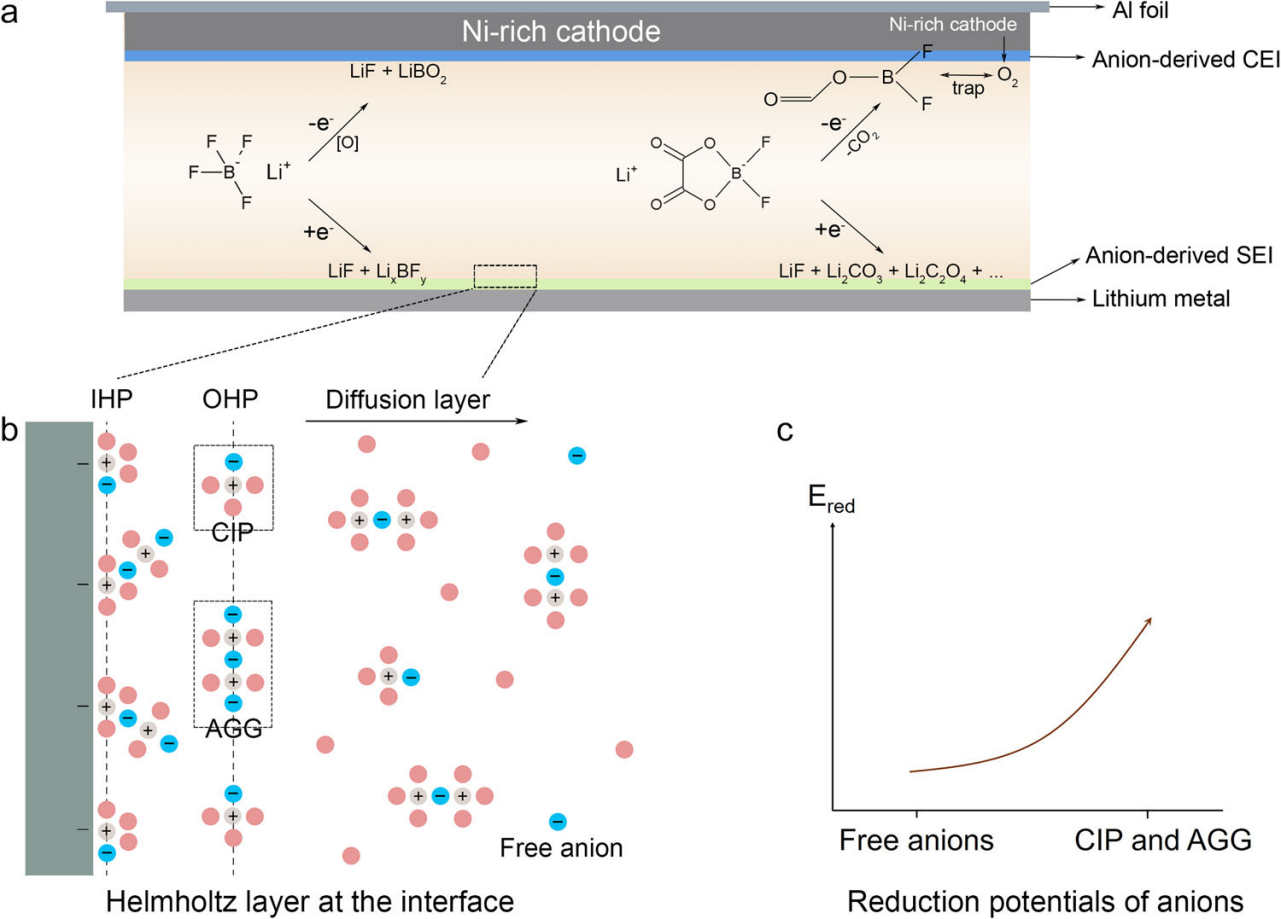
Design principle of the anion-enrichment interface. Schematic illustration of **a** anion-enrichment interface and anion-derived EEIs and **b** electrode interface including inner Helmholtz plane (IHP), outer Helmholtz plane (OHP), and diffusion layer. CEI and SEI refer to the cathode and solid-electrolyte interphase, respectively. CIP and AGG refer to contact ion pair and aggregate, respectively. Pink, gray, and blue balls represent solvents, lithium ions, and anions, respectively. **c** Reduction potentials of free anions, CIP and AGG.

To decipher how the anion-enrichment interface dictates the interfacial chemistry, it is the priority to understand the structure of the inner Helmholtz plane (IHP) that is the locus of the electrical center of adsorbed ions and strongly associated with the formation of the EEI^56^. In our proposed WSDE electrolyte, driven by the electric field, Li^+^, accompanied by anions in the primary solvation sheath (CIP and AGG), transports from the bulk electrolyte to the IHP of the electrode surface (Fig. 1b). Anions in IHP are prone to decompose to form an anion-derived dense EEIs (Fig. 1a and Supplementary Fig. 1a) and exclude solvents from the direct contact with the electrode. In contrast in the traditional electrolyte, Li^+^ is mainly separated by solvents with anions expelled from the primary solvation sheath (Supplementary Fig. 2). Solvated Li^+^ brings solvents into the IHP generating the solvent-derived porous EEIs (Supplementary Fig. 1b). The classic molecular dynamics (cMD) simulations are performed to profile the ion distribution at the electrode/electrolyte interface (Supplementary Figs. 3 and 4). When no charge is applied to the electrode, neither Li^+^ nor anions (indicated by B) dominate the electrode/electrolyte interface. The ions tend to evenly distribute in the bulk electrolyte and the interface. When the charge of 5 μC cm^−2^ is applied, the distribution of Li^+^ and anions at the electrode/electrolyte interface is highly correlated, indicating that in our electrolyte attracted by negative charge Li^+^ accompanied by anions migrates to the interface forming an anion-enrichment interface. Also, ions and charges are closer to the Cu electrode, beneficial for them to take part in the interface reaction. When a higher charge of 10 μC cm^−2^ is applied, ion enrichment at the electrode/electrolyte interface increases. Besides, in the WSDE electrolyte, the reduction potential of anions is also altered by the intimate interaction with Li^+^. The high degree of Li-anion association will remarkably promote the onset of the reduction potential of Li-anion complex^54,57^, contributing to the anion-derived EEIs (Fig. 1c and Supplementary Fig. 5). Therefore, the anion-enrichment interface and thus elevated reduction potential of Li-anion complex synergistically enhance the formation of anion-derived EEIs, promising the long-cycling LMA and Ni-rich cathode.

### Characterizations and simulation of the solvation structure

**Fig. 2:**
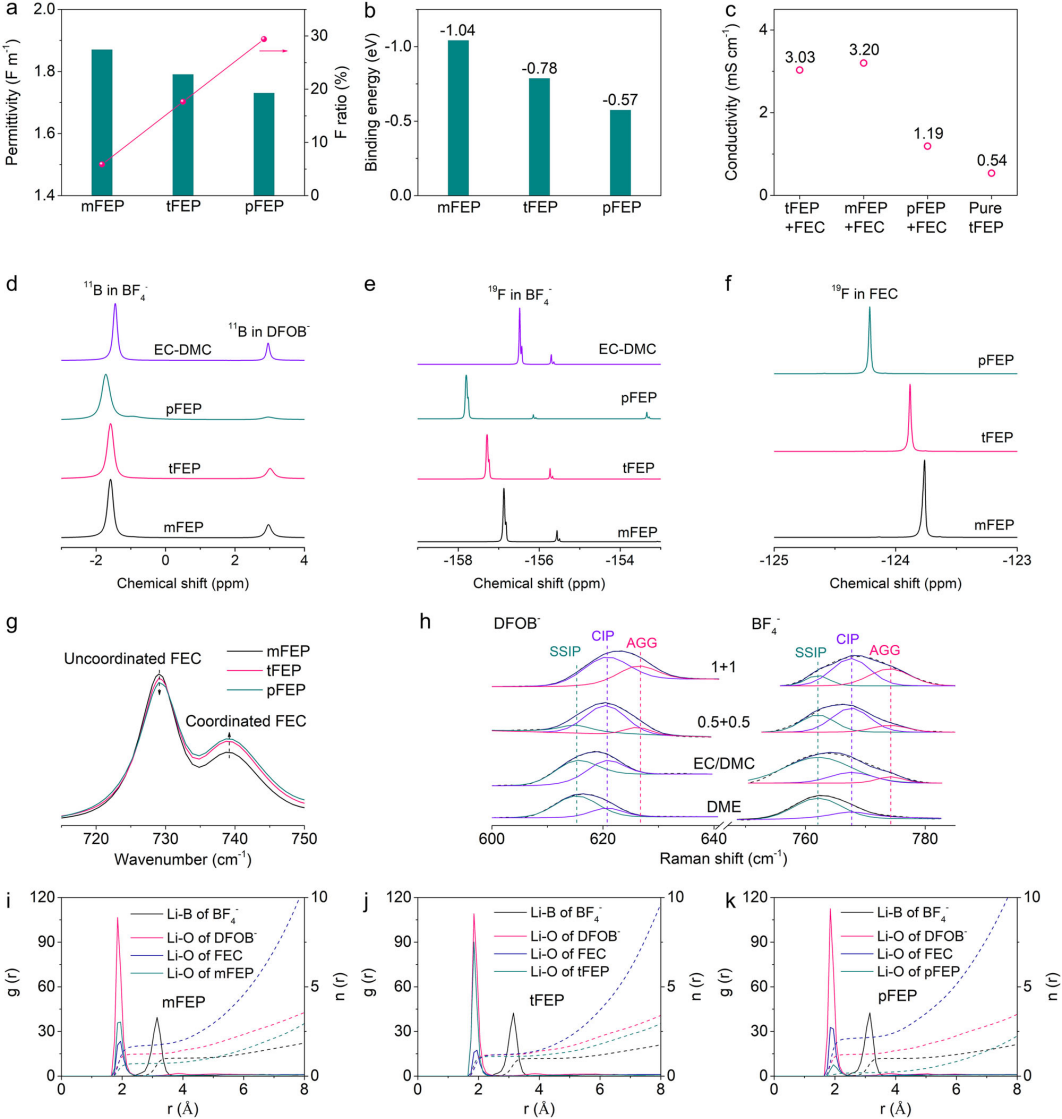
Evolution and simulation of the solvation structures. **a** Permittivity and fluorine ratio of FEP-based solvents (mFEP, tFEP, and pFEP). **b** Molecular dynamic (MD) simulation results of the binding energy between a Li-ion and solvent in a carbonate oxygen site. **c** The conductivity of several electrolytes: 1 M LiBF_4_ + 1 M LiDFOB in tFEP/FEC, mFEP/FEC, pFEP/FEC, and pure tFEP electrolytes. **d** ^11^B of BF_4_^−^ and DFOB^−^, ^19^F of **e** BF_4_^−^ and **f** FEC NMR spectra in 1 M LiBF_4_ + 1 M LiDFOB mFEP/FEC, tFEP/FEC, pFEP/FEC, and EC/DMC electrolyte. **g** FT-IR spectra of C=O breathing vibration band for FEC in 1 M LiBF_4_ + 1 M LiDFOB FEP/FEC electrolyte. **h** Raman spectra for DFOB^−^ and BF_4_^−^ anions with LiDFOB and LiBF_4_ dissolved in various solvents. 1 + 1 and 0.5 + 0.5 refer to 1 M LiBF_4_ + 1 M LiDFOB tFEP/FEC and 0.5 M LiBF_4_ + 0.5 M LiDFOB tFEP/FEC electrolyte, respectively. Radical distribution functions (RDFs, g(r), solid) and cumulative distribution functions (CDFs, n(r), dashed line) of boron and oxygen around Li^+^ as a function of distance (r) from MD simulations in **i** mFEP-, **j** tFEP-, and **k** pFEP-based electrolyte.

To target an anion-enrichment interface and solvation structure, the solvating power of solvents and the dissociation constant of salts are carefully manipulated. Compared with linear carbonate esters, ethyl propionate (EP), as a typical linear carboxylic ester, has higher oxidation stability, stronger binding with Li^+^, and better compatibility with LMA^30,58,59^, and thus is proposed as the base solvent in our electrolyte. Besides, the –F group is introduced to customize the solvating power and facilitate the LiF-abundant interphasial chemistry. The more fluorine substituent, the lower solvating power of solvents^60,61^. When the mono –F group is introduced, ethyl 2-monofluoropropionate (mFEP) with relatively high permittivity (1.87) is not in favor of LMA (Fig. 2a). Increasing –F groups to five will render ethyl pentafluoropropionate (pFEP) with ultralow permittivity, incapable of dissolving enough lithium salts or guaranteeing enough ionic conductivity of electrolyte (Fig. 2c). While ethyl 3,3,3-trifluoropropanoate (tFEP) with three –F substituents has the appropriate permittivity of 1.79, which is compatible with Li metal and meanwhile supports the sufficient ionic conductivity. The permittivity of these three solvents is in accord with the chemical shifts of ^23^Na NMR measurements (Supplementary Fig. 7). The binding energy between a Li-ion and solvents is also calculated using quantum chemistry calculations to confirm the permittivity, in which the binding energy decreases in the order of mFEP > tFEP > pFEP (Fig. 2b and Supplementary Fig. 8, Supplementary Tables 2 and 3). Accordingly, tFEP is used as the main solvent in our work, because of the optimal permittivity, solvating power, and fluorine substituent, as well as sufficient ionic conductivity.

Besides solvents, lithium salts are also screened to obtain the optimum dissociation constant (Supplementary Fig. 9). In dilute electrolytes, dissolution primarily involves the solvation of Li ions with a competition present between solvents and anions for coordination to Li^+^ cation. The solvation number can be leveraged to evaluate the dissociation constant^62–64^. LiPF_6_, LiFSI, and LiTFSI with high solvation numbers result in the anions hardly competing with solvents to coordinate with Li ions. While LiBF_4_ and LiDFOB with low solvation numbers are chosen as the main salts in this work because the weak dissociation facilitates the formation of the anion-rich solvation structure^30,55^. Besides, the reduction of LiBF_4_ and LiDFOB will form robust SEI to support long-cycling LMA^65^, in addition to LiDFOB enhancing the interfacial stability of high-voltage Ni-rich cathodes (Supplementary Fig. 6)^66,67^. Conductivity measurements are also conducted to indicate the dissociation constant (Supplementary Fig. 10), in which higher conductivity manifests that the salts are more readily dissociated. LiBF_4_ and LiDFOB with modest conductivity have the appropriate dissociation constants in accord with the solvation number (Supplementary Fig. 9). In this work we combine dual solvents of tFEP and FEC with optimal permittivity and solvating power with weakly dissociated LiBF_4_ and LiDFOB to prepare WSDE electrolyte (1 M LiBF_4_ + 1 M LiDFOB in tFEP/FEC). FEC herein is introduced to support a high ionic conductivity (3.03 mS cm^−1^ vs. 0.54 mS cm^−1^ for pure tFEP-based electrolyte, Fig. 2c).

The evolution of solvation structures with the solvating power of solvents is studied with spectroscopies, in which LiBF_4_ and LiDFOB are dissolved into various FEP-based solvents with EC/DMC as the reference solvent. Nuclear magnetic resonance (NMR) is adopted to probe the ion-solvent interaction of electrolytes. The chemical shift of ^11^B nuclei in BF_4_^−^ anion shows a red shift in the order of EC/DMC > mFEP > tFEP > pFEP (Fig. 2d)^68–70^, indicating that the affinity of BF_4_^−^ with Li^+^ becomes increasingly strong, which can be confirmed by the similar trend in chemical shift of ^19^F nuclei in BF_4_^−^ (Fig. 2e)^68,70^. Following the same order, ^11^B nuclei of both BF_4_^−^ and DFOB^−^ display obvious peak broadening, due to the increasingly intimate interaction between anions and Li and thus the semisolid-state anion environment^57^. The chemical shift of ^19^F nuclei in FEC decrease in the order of mFEP > tFEP > pFEP (Fig. 2f)^71^, signifying that the affinity between FEC and Li^+^ follows the same trend whilst the interaction between FEP-based solvents and Li^+^ gradually attenuates. The solvation structure is further consolidated by FT-IR spectra, in which C=O breathing vibration bands for FEC display two peaks at 729 and 739 cm^−1^ (Fig. 2g)^72^, corresponding to the uncoordinated and coordinated FEC, respectively. Along with the decrease of solvation power from mFEP to pFEP, the intensity of coordinated FEC enhances^73^, indicative of more FEC bonding with Li^+^. The C=O stretching mode of FEP at 1734 cm^−1^ further confirms that mFEP and tFEP are coordinated with Li^+^. Whilst no such a peak appears in the pFEP-based electrolyte, which can be interpreted by weak interaction between Li^+^ and pFEP with ultralow solvating power (Supplementary Fig. 11)^74^.

The solvation structure is further disclosed by Raman spectra. As the salts concentration elevates, the vibration of –CH_2_–CO_2_ out-of-plane bending band of tFEP (664.6 cm^−1^)^75^ (Supplementary Fig. 12) and ring deformation of FEC (726.7 cm^−1^)^76^ (Supplementary Fig. 13) drift upwards, denoting that more solvents bond with Li with free solvents lessening^77–79^. The symmetric B–F stretching Raman band in BF_4_^−^ anion can be deliberately deconvolved into three components: free anion (FA, 761.94 cm^−1^, non-coordinated BF_4_^−^), contact ion pair (CIP, 767.7 cm^−1^, one BF_4_^−^ bonding with one Li^+^), and ion aggregate (AGG, 774.6 cm^−1^, one BF_4_^−^ bonding with two or more Li^+^) (Fig. 2h)^80–82^. The fractions of band area are calculated, in which 1 M LiBF_4_ + 1 M LiDFOB in tFEP/FEC electrolyte contains 12.2% of FA, 52.5% of CIP, and 35.3% of AGG, indicating that the solvation structure is dominated by CIP and AGG (Supplementary Fig. 14a). The proportion of CIP and AGG increases with the reduction of solvating power in the order of DME > EC/DMC > tFEP/FEC. Also, the higher concentration favors the access of anions into the primary solvation sheath generating more CIP and AGG. Similar results are obtained in DFOB^−^ anions (Supplementary Fig. 14b). Accordingly, weakly dissociated salts facilitate anions to expel solvents with low solvation power from the primary solvation sheath that therefore is dominated by CIP and AGG, leading to the formation of anion-rich solvation structure.

The microscopic structure of solvated Li^+^ in various electrolytes is deciphered by calculating the RDFs and CDFs using molecular dynamics (MD) simulations (Fig. 2i–k). The RDFs show that both solvents (FEC and FEP) and anions (BF_4_^−^ and DFOB^−^) involve the formation of Li^+^ primary solvation sheath. DFOB^−^ anions are strongly packed around Li^+^ with the first peak position at approximately 1.85 Å, while BF_4_^−^ anions are farthest from Li^+^ with the first RDF peaks of 3.15 Å. Unlike mFEP (Fig. 2i) and pFEP (Fig. 2k), tFEP, next only to DFOB^−^, is tightly bonded with Li^+^ (Fig. 2j), denoting that tFEP, as the main solvent, will intensely determine the solvation structure of tFEP-based electrolyte. The coordination numbers within the first sheath are estimated from CDF curves at the location of the first valley of the RDF (3.75 Å), in which FEC dominates in the mFEP- and pFEP-based electrolyte, representative of the traditional solvent-rich solvation structure. In contrast, the coordination number of anions (DFOB^−^ + BF_4_^−^: 2.28) is comparable to solvents (FEC + tFEP: 2.48) in tFEP-based electrolytes (Supplementary Fig. 15), indicating that plenty of anions appear in the first sheath coordinating with Li^+^. MD simulations are in accord with characterization results corroborating the anion-rich solvation structure in 1 M LiBF_4_ + 1 M LiDFOB tFEP/FEC electrolyte.

### SEI chemistry of LMA

**Fig. 3:**
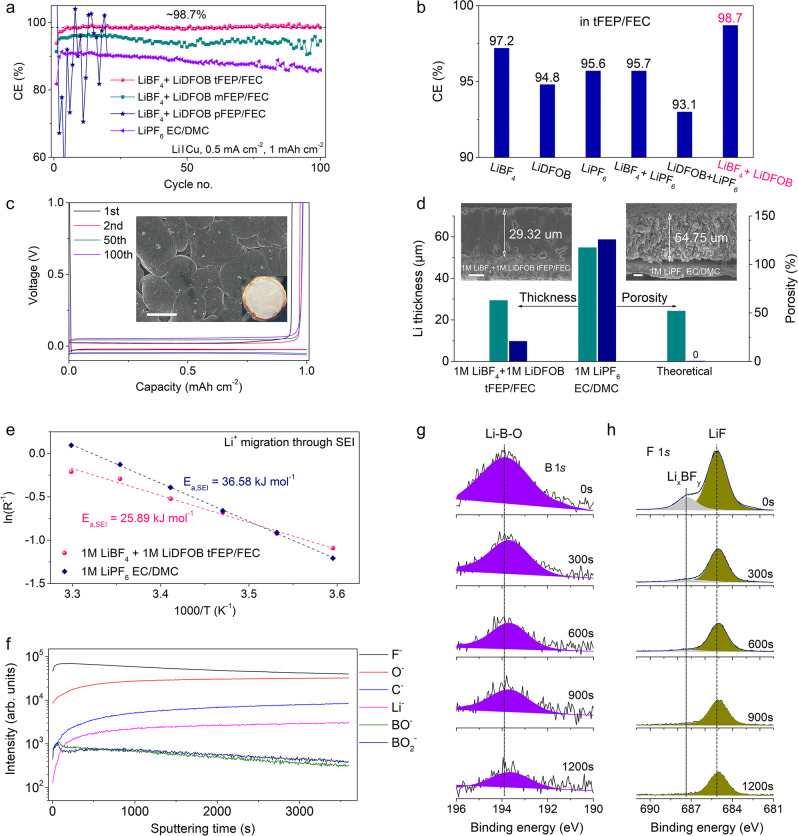
Electrochemical performance and characterizations of LMA. **a**, **b** Coulombic efficiency for Li deposition/stripping in Li/Cu half cells using various electrolytes at the current density of 0.5 mA cm^−2^ for the capacity of 1 mAh cm^−2^. **c** The corresponding deposition/stripping curves in 1 M LiBF_4_ + 1 M LiDFOB tFEP/FEC electrolyte. Inset is the top view of Li morphology deposited on Cu foil. Scale bar: 10 μm. **d** Electrode thickness and porosity of deposited Li calculated from focused ion beam-scanning electron microscopy (FIB-SEM) results. Inset are cross-section SEM images of Li metal deposited on Cu at the current density of 0.5 mA cm^−2^ for 10 h in 1 M LiBF_4_ + 1 M LiDFOB tFEP/FEC and 1 M LiPF_6_ EC/DMC electrolyte. Scale bar: 10 μm. **e** Arrhenius plot for the resistance of Li^+^ migration through SEI. **f** Time-of-flight secondary ion mass spectrometry (TOF-SIMS) depth profiles of various chemical species for LMA in 1 M LiBF_4_ + 1 M LiDFOB tFEP/FEC electrolyte. XPS spectra of **g** B 1*s* and **h** F 1*s* for LMA in 1 M LiBF_4_ + 1 M LiDFOB tFEP/FEC electrolyte after various sputtering times: 0, 300, 600, 900, and 1200 s.

To uncover the effect of solvation structure on interfacial chemistry, the electrochemical performance of LMA is investigated using Li/Cu half cells. The 1 M LiBF_4_ + 1 M LiDFOB tFEP/FEC electrolyte delivers an average CE of 98.7% for Li stripping/plating within 100 cycles, much higher than that of mFEP-based electrolyte (below 96%) and 1 M LiPF_6_ EC/DMC baseline electrolyte (~89%) (Fig. 3a). In contrast, the pFEP-based electrolyte cannot support a stable Li cycle probably due to its low conductivity of 1.19 mS cm^−1^ (Fig. 2c). To highlight the advantages of dual salts of LiBF_4_ + LiDFOB, the electrolytes were prepared using various salts in tFEP/FEC solvents. The CE of LiBF_4_ in tFEP/FEC electrolyte slowly climbs to ~97.2% within 100 cycles, while LiDFOB- and LiPF_6_-based electrolytes hardly endure a long cycle (Fig. 3b and Supplementary Fig. 16a–c). The typical Li deposition/stripping curves of 1 M LiBF_4_ + 1 M LiDFOB tFEP/FEC electrolyte show a low overpotential of ~20 mV with the marginal increase within 100 cycles (Fig. 3c). In contrast, other electrolytes exhibit the constantly growing overpotential and unstable cycle (Supplementary Fig. 17), likely due to the continuous crack/reconstruction of SEI and the formation of “dead Li”, respectively.

The morphology and interfacial chemistry of deposited Li are carefully characterized. In contrast to the highly porous and whisker-like Li in 1 M LiPF_6_ EC/DMC baseline electrolyte (Supplementary Fig. 18), Li deposits in 1 M LiBF_4_ + 1 M LiDFOB tFEP/FEC electrolyte are notably close-packed (Fig. 3c) and bond firmly with Cu substrate (Supplementary Fig. 19a). After 100 cycles, large amounts of SEI and dead Li are left in the Cu foil after Li stripping in baseline electrolyte, while in our electrolyte Cu foils maintain smooth with no observable dead Li (Supplementary Fig. 20). The contrast in cross-section morphology of Li deposits obtained from focused ion beam-scanning electron microscopy (FIB-SEM) is even more noticeable. In 1 M LiBF_4_ + 1 M LiDFOB tFEP/FEC electrolyte, Li deposits with a capacity of 5 mAh cm^−2^ at 0.5 mA cm^−2^ are dense and seamlessly packed on the Cu substrate forming perfect columnar structures with negligible holes and the thickness of 29.32 μm (Fig. 3d). By comparison in 1 M LiPF_6_ EC/DMC electrolyte, highly porous Li deposits grow in an uncontrolled fashion with the thickness of 54.75 μm. The weak bond leads to a clear crack between Li deposits and Cu substrate (Fig. 3d) and thus some Li deposits are stripped from the Cu substrate and adhered to the separator in disassembled cells (Supplementary Fig. 19b, d). Ideally, the deposited Li (5 mAh cm^−2^) should exhibit a theoretical thickness of 24.25 µm with zero porosity. The thicknesses of 29.32 and 54.75 μm in 1 M LiBF_4_ + 1 M LiDFOB tFEP/FEC and 1 M LiPF_6_ EC/DMC electrolyte correspond to the porosities of 20.91 and 125.77%, respectively (Fig. 3d). The lower porosity of Li deposits in 1 M LiBF_4_ + 1 M LiDFOB tFEP/FEC electrolyte will diminish the surface exposure to the liquid electrolyte, alleviating extra parasitic reactions that consume the electrolyte and active Li and in favor of high CE for Li stripping/deposition.

To elucidate the rationale of different Li deposits morphologies, the rate-determining steps of Li deposition are investigated (Fig. 3e and Supplementary Fig. 21). The activation energy for Li^+^ transport through SEI in 1 M LiBF_4_ + 1 M LiDFOB tFEP/FEC electrolyte (*E*_a, SEI_ = 25.89 kJ mol^−1^) is remarkably lower than that in 1 M LiPF_6_ EC/DMC electrolyte (*E*_a, SEI_ = 36.58 kJ mol^−1^). Higher *E*_a, SEI_ indicates the slow diffusion of Li ions through SEI, which renders insufficient Li ions beneath SEI preferentially gathering on the tip of nucleation sites and thus forming dendritic Li deposition in 1 M LiPF_6_ EC/DMC electrolyte^83–86^. In contrast, fast diffusion of Li-ion through SEI in 1 M LiBF_4_ + 1 M LiDFOB tFEP/FEC electrolyte affords abundant Li-ion beneath the SEI. In this case, Li-ion obtains electrons at each site of the Li nucleation bumps forming close-packed Li deposits^87^.

The migration of Li-ion through SEI is closed related to the chemical composition and structure of SEI that is examined via time-of-flight secondary ion mass spectrometry (TOF-SIMS) with the spatially resolving nature (Fig. 3f and Supplementary Fig. 22) and X-ray photoelectron spectroscopy (XPS) with an Ar^+^ sputtering depth profiling (Fig. 3g, h and Supplementary Figs. 23–26). The depth profiles of intensity profiles of the representative fragments generated by Ga-ion sputtering show that F^−^ and O^−^ are dominant in 1 M LiBF_4_ + 1 M LiDFOB tFEP/FEC electrolyte, whilst the intensity of C^−^, denoting the organic species, is an order of magnitude weaker (Fig. 3f). Besides, the intensity of BO^−^ and BO_2_^−^, only from the reduction of anions, is readily observed. Such a SEI structure in 1 M LiBF_4_ + 1 M LiDFOB tFEP/FEC electrolyte can be visualized by 3D reconstruction (Supplementary Fig. 22). According to XPS results, the top surface of the SEI consists of both organic (COOR, C–O) and inorganic (LiF, Li_2_O) components (Fig. 3g, h and Supplementary Figs. 23 and 24). After sputtering for 300 s in 1 M LiBF_4_ + 1 M LiDFOB tFEP/FEC electrolyte, the C 1*s* signal, indicative of organic decomposition products, dramatically drops to the noise level (Supplementary Figs. 23a and 26a), accompanied by LiF signal maintaining strong throughout the whole sputtering process (Fig. 3h). More worth mentioning that the B 1*s* signal can be observed even after the 1200 s sputtering (Fig. 3g), indicating that the reduction of anions (BF_4_^−^ and DFOB^−^) plays an important role in constructing SEI. In contrast in 1 M LiPF_6_ EC/DMC baseline electrolyte, the C 1*s* signal persists after sputtering (Supplementary Figs. 23b and 26b). P 2*p* signal dramatically drops to the noise level after 300 s sputtering (Supplementary Fig. 25), signifying that PF_6_^−^ is scarcely reduced in the baseline electrolyte.

From the results of TOF-SIMS and XPS, different from the mosaic model of SEI in 1 M LiPF_6_ EC/DMC baseline electrolyte, the SEI in 1 M LiBF_4_ + 1 M LiDFOB tFEP/FEC electrolyte is mainly composed of inorganic components. The compact and robust SEI has a high Young’s modulus to effectively suppress the growth of lithium dendrites and thus enable high safety and long lifespan of LMBs^36,37^. Besides, the inorganic-abundant SEI will create abundant phase boundaries and vacancies to facilitate Li^+^ diffusion and cut down the activation energies of interphasial processes. The uniform and rapid diffusion of Li ions through SEI will tailor the formation of dense and close-packed Li deposits^88,89^. The robust and fast Li^+^-conductive SEI and thus closely-packed Li deposits jointly guarantee the high CE and long lifespan of LMA.

### Performance of 4.6 V NCM811 and LiCoO_2_ cathode

**Fig. 4:**
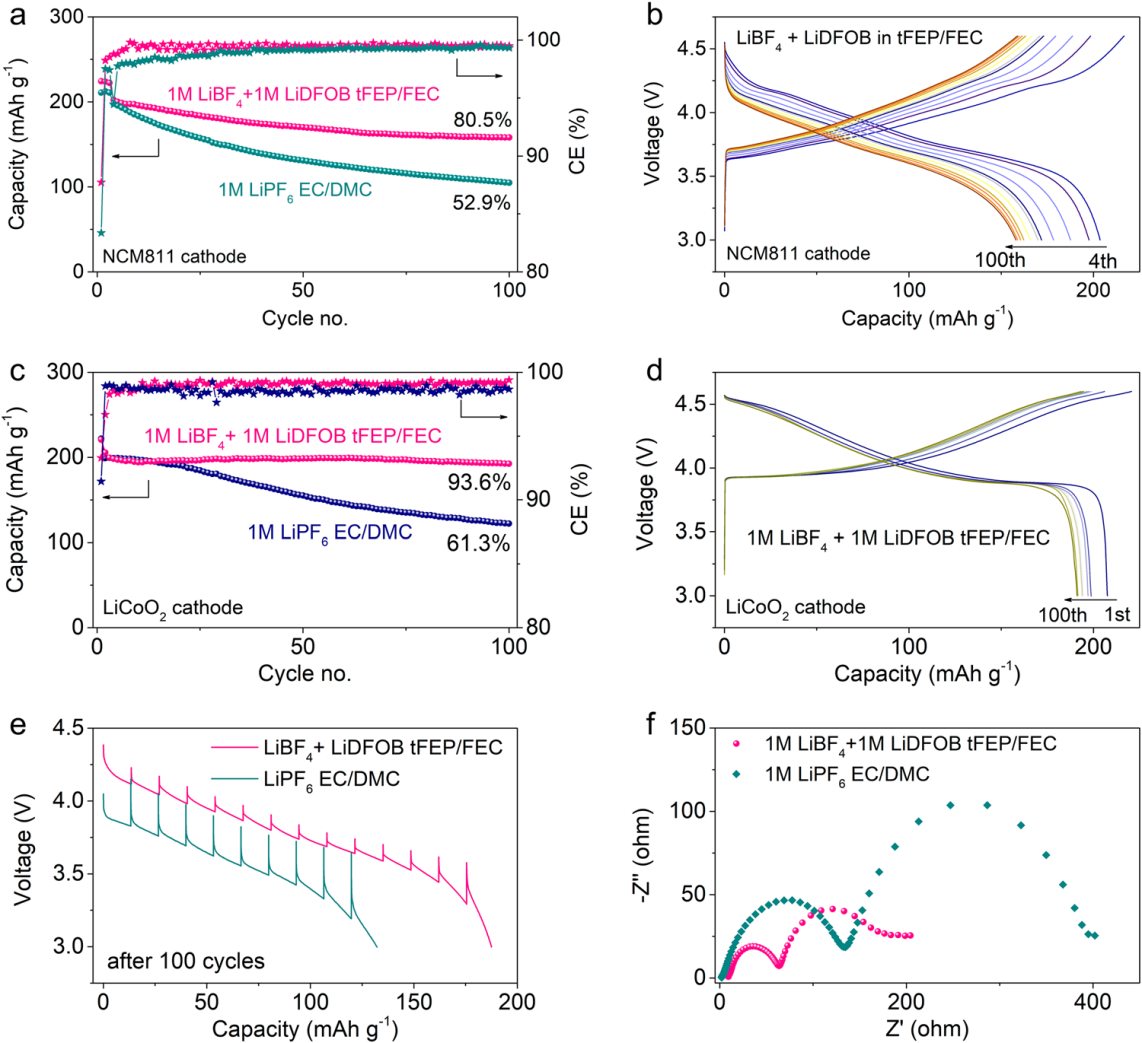
Electrochemical performance of 4.6 V NCM811 and LiCoO_2_ cathode. Cycling performance and CE of **a** NCM811 cathode and **c** LiCoO_2_ at 0.5 C rate (0.2 C for the first three cycles, 1C = 200 mA hg^−1^). Corresponding voltage profiles of **b** NCM811 and **d** LiCoO_2_ cathode in 1 M LiBF_4_ + 1 M LiDFOB tFEP/FEC electrolyte. **e** Discharge voltage profiles of galvanostatic intermittent titration technique measurements (GITT) after 100 cycles. The cells were discharged for 20 min at 0.2C followed by relaxation for 1 h. **f** Nyquist spectra of NCM811 cathodes after 100 cycles. The impedance of NCM811 electrodes was monitored using a 3-electrode cell to exclude the impact of the Li anode.

The compatibility of 1 M LiBF_4_ + 1 M LiDFOB tFEP/FEC electrolyte with high-voltage cathodes was evaluated using aggressive 4.6 V NCM811 and LiCoO_2_ cathode (Fig. 4). A high capacity of 224 mAh g^−1^ is delivered at 0.2 C when NCM811 cathode is charged to 4.6 V (Fig. 4a). 80.5% of the capacity maintains after 100 cycles with CEs approaching ~99.6%. In contrast, only 52.9% of the capacity remains in 1 M LiPF_6_ EC/DMC baseline electrolyte with low CEs of less than 99%. Also, our electrolyte exhibits more stable voltage profiles and higher energy efficiency (Fig. 4b) compared to the baseline electrolyte (Supplementary Fig. 27). The large voltage drop at the beginning of the discharge process can be primarily ascribed to overpotentials from NCM811 cathode degradation and increasing SEI thickness of Li metal anode over cycling. Besides the NCM811 cathode, our electrolyte is well compatible with a 4.6 V LiCoO_2_ cathode. The capacity of 205.6 mAh g^−1^ is obtained with a retention of 93.6% and no observable voltage decay after 100 cycles in our electrolyte (Fig. 4c, d). In contrast, only 61.3% of the capacity remains in 1 M LiPF_6_ EC/DMC baseline electrolyte (Fig. 4c and Supplementary Fig. 30).

The rate capability of NCM811 cathode in our electrolyte exhibits high capacities of 190 and 163 mAh g^−1^ at 1C and 2C, respectively (Supplementary Fig. 31a), readily outperforming the baseline electrolyte (Supplementary Fig. 31b). Also, the voltage hysteresis of the discharge process in our electrolyte is much less than that in the baseline electrolyte (Supplementary Fig. 31). The underlying rationale is investigated by galvanostatic intermittent titration technique (GITT) measurements (Fig. 4e). Despite similar overpotentials at the 1st cycle (Supplementary Fig. 32), the overpotentials after 100 cycles are much higher in baseline electrolyte than those in our electrolyte, which might be attributed to the phase transition, structural damage, and more resistive interphasial layers^20^. The interphasial resistance was investigated using electrochemical impedance spectroscopy (EIS) measurements in 3-electrode cells to exclude the impact of LMA (Fig. 4f). After 100 cycles, the resistances of CEI (the semicircle at high frequency) and charge-transfer process (the semicircle at the medium frequency) in our electrolyte are much lower than those in baseline electrolyte.

### Cathode chemistry of 4.6 V NCM811

**Fig. 5:**
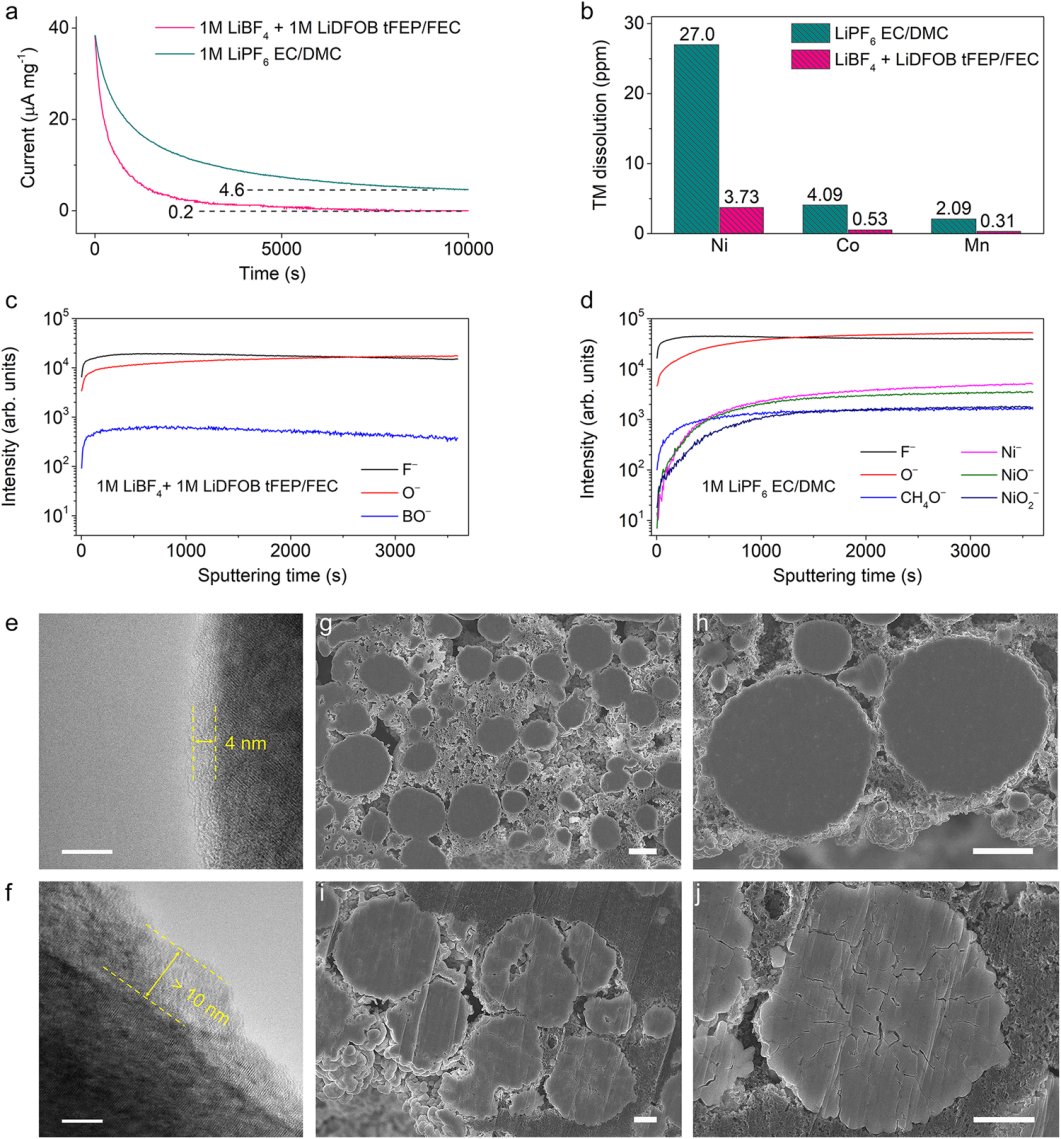
Characterizations of cathode chemistry with 4.6 V cut-off voltage in 1 M LiBF_4_ + 1 M LiDFOB tFEP/FEC and 1 M LiPF_6_ EC/DMC electrolyte. **a** Leakage currents during the 4.6 V constant-voltage floating test of the NMC811 cathodes cycled in various electrolytes for 3 cycles. **b** Transition metal (TM) dissolution measured by inductively coupled plasma mass spectrometry (ICP-MS) after 100 cycles. TOF-SIMS characterization of the cycled NCM811 cathodes retrieved from the pouch cells after 100 cycles. Normalized depth profiles of interphasial and bulk fragments, illustrating the structure of CEIs in **c** 1 M LiBF_4_ + 1 M LiDFOB tFEP/FEC and **d** 1 M LiPF_6_ EC/DMC electrolyte. High-resolution TEM images of NCM811 interphases after 100 cycles in **e** 1 M LiBF_4_ + 1 M LiDFOB tFEP/FEC and **f** 1 M LiPF_6_ EC/DMC electrolyte. Scale bar: 10 nm. SEM images of cross-sectioned NMC811 cathodes cycled in 1 M LiBF_4_ + 1 M LiDFOB tFEP/FEC (**g**, **h**) and 1 M LiPF_6_/EC-DMC (**i**, **j**) electrolytes. Scale bar: 5 μm (**g**, **h**) and 2 μm (**i**, **j**).

The cathode interfacial chemistry, closely related to the NCM811 performance, was extensively investigated (Fig. 5). The side reactions between the cathode and electrolyte were firstly pinned down using accelerated degradation tests by persistently exposing the cathode at 4.6 V, in which the float-test leakage current denotes the side reaction rates (Fig. 5a). The leakage current in baseline electrolyte slowly decreases to ~4.6 μA mg^−1^ in the 10,000 s hold. In contrast, the leakage current in our electrolyte fleetly decays to a minimum value of 0.2 μA mg^−1^, indicative of a pale reaction rate and stabilized interphasial chemistry. The transition metals (TMs) dissolution playing a vital role in cathode degradation was studied by inductively coupled plasma mass spectrometry (ICP-MS) measurements. After 100 cycles, the dissolved TMs is prominently less in our electrolyte in comparison with those in the baseline electrolyte (Fig. 5b). As the dissolved TMs will eventually waft to the anode side leading to the destruction of SEI, the marginally dissolved TMs in our electrolyte considerably mitigate the cross-over effect (Supplementary Fig. 34 and Tables 4 and 5), which will contribute to the cycling stability of LMBs.

To gain insight into the low resistance in 1 M LiBF_4_ + 1 M LiDFOB tFEP/FEC electrolyte, the interfacial component of NCM811 cathode after cycling was characterized. TOF-SIMS with an ultrahigh chemical selectivity and surface sensitivity was performed on the cycled NCM811 cathode. The depth profiles of some secondary ion fragments show that CEI in our electrolyte mainly consists of inorganic F-, O-, and BO- segments (Fig. 5c and Supplementary Fig. 35), which is in accord with the XPS results (Supplementary Fig. 37). These components form a dense and robust CEI to restrain the parasitic reaction between NCM811 cathode and the electrolyte. Whilst the CEI in baseline electrolyte is mainly composed of F-, O-, CH_4_O-, Ni-, NiO-, and NiO_2_- (Fig. 5d and Supplementary Fig. 36). The CH_4_O- segment is probably from the ring opening reaction of EC, indicative of the serious detrimental oxidation reactions of EC-based electrolyte during cycling. NiO_2_- segment represents the bulk NCM811 cathode, while NiO- and Ni- segments can be ascribed to the rock-salt phase from phase transformation and Ni dissolution, respectively. In contrast, no TM segments are observed in our electrolyte, illustrating that the inorganic-abundant CEI considerably restrains the parasitic reaction and prohibits TM dissolution. For the XPS O 1*s* spectra (Supplementary Fig. 37c), no signal of metal oxide bonds (M–O) is observed at ~530 eV in the 1 M LiBF_4_ + 1 M LiDFOB tFEP/FEC electrolyte, indicating that the electrolyte can effectively passivate the highly active cathode interface.

The CEI of cycled NCM811 was directly observed using high-resolution transmission electron microscopy (HRTEM). The NCM811 cycled in our electrolyte is coated by amorphous CEI with a thickness of ~4 nm (Fig. 5e). The layered structure near the surface is maintained well with no observable phase transition. In contrast, the cathode interface in the baseline electrolyte exhibits a clear surface degradation with a readily observed rock-salt layer of more than 10 nm (Fig. 5f). These findings indicate that our electrolyte greatly mitigates the cathode surface degradation and resultantly suppresses the phase transformation over cycling. In addition to acting on the surface, the CEI formed in our electrolyte contributes to restraining the cracking of NCM811 secondary particles. Intergranular cracking between connected primary particles is a critical issue for the degradation of high-voltage Ni-rich cathodes, leading to the loss of electrical contact between primary particles. The resultant high surface area means more liquid electrolytes required for wetting, severe side reactions, and massive electrolyte consumption. The intergranular cracking was further inspected using SEM on the cross-sectioned NCM811 cathodes after 100 cycles. In sharp contrast to the apparent cracking in the baseline electrolyte (Fig. 5i, j and Supplementary Fig. 38c, d), the microstructural degradation of NCM811 cathode is remarkably suppressed in our electrolyte (Fig. 5g, h and Supplementary Fig. 38a, b).

Upon electrochemical cycles, the repetitive delithiation/lithiation process induces constant volume expansion and contraction, which generates mechanical stresses that facilitate the formation of defects concentrated at the grain boundaries. The generation of nano- or micro-defects does not necessarily cause the disintegration of secondary particles or macroscopic damages^90^. However, in baseline electrolytes, the continuous chemical corrosion to the cyclically slipping grain boundaries of the highly-charged NCM811 cathode offers thermodynamic and kinetic advantages to the initiation and propagation of intergranular cracking and deteriorates the cathodes. While in our electrolyte the uniform and dense CEI maintains well enduring the repetitive delithiation/lithiation process, which protects the NCM811 cathode from the continuous chemical corrosion safely circumventing the intergranular cracking.

### Anode-free Cu/NCM811 pouch cells

**Fig. 6:**
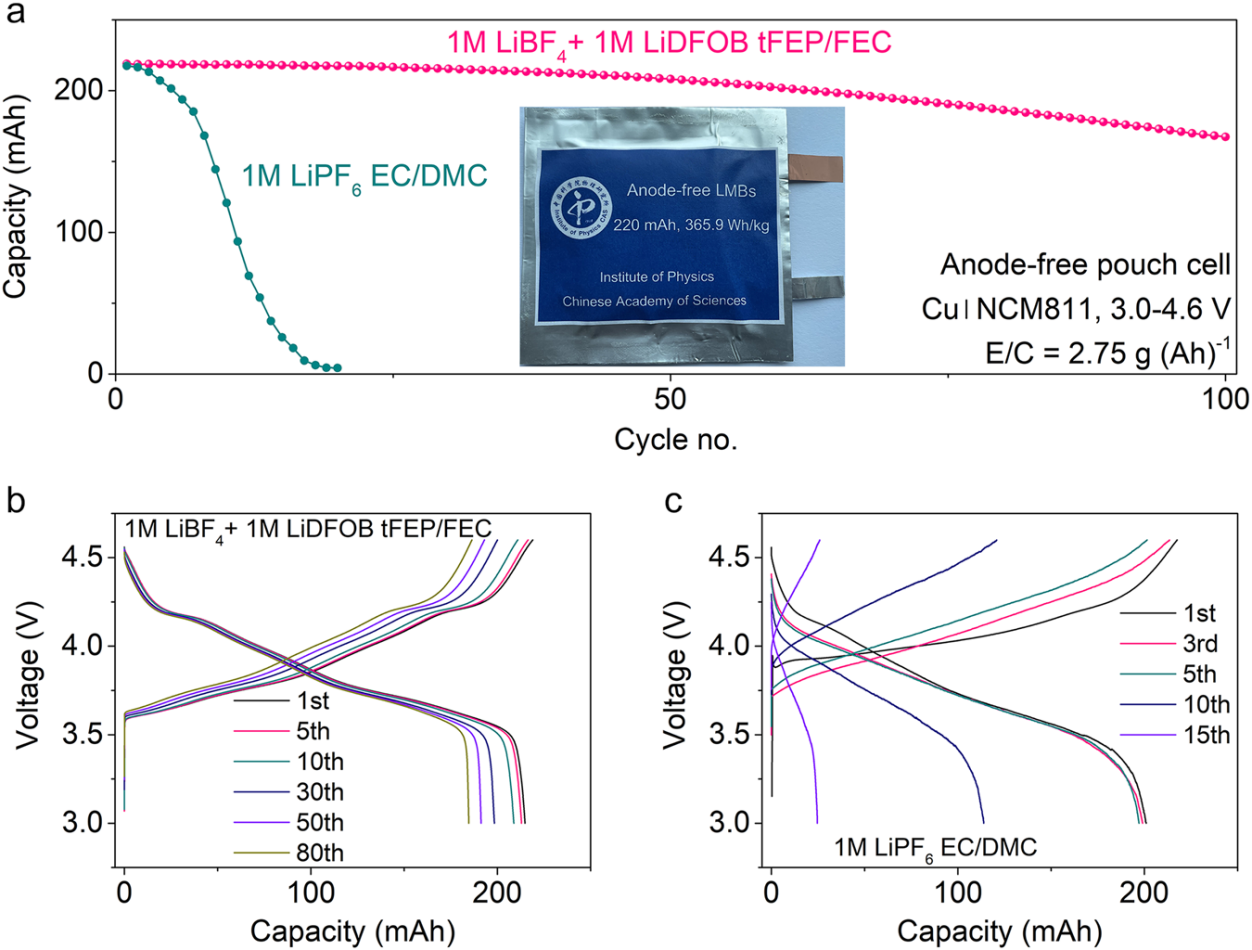
Electrochemical performance of anode-free Cu|NCM811 pouch cells. **a** Cycling performance and corresponding voltage profiles using **b** 1 M LiBF_4_ + 1 M LiDFOB tFEP/FEC and **c** 1 M LiPF_6_ EC/DMC electrolyte at 0.1C charge and 0.5C discharge between 3.0 and 4.6 V with electrolyte absorbance of 2.75 g (Ah)^−1^.

To systematically highlight the compatibility of our electrolyte with LMA and 4.6 V NCM811 cathode, aggressive anode-free pouch cells were constructed with harsh conditions of zero-excess lithium, high cathode loading of 4.64 mAh cm^−2^, lean electrolyte absorbance of 2.75 g (Ah)^−1^. The two-layer pouch cells with a high capacity of 220 mAh, corresponding to the energy density of 365.9 Wh/kg (Supplementary Table 6), can achieve a capacity retention of 80% after 100 cycles at 0.1C charge and 0.5C discharge rate in our electrolyte (Fig. 6a, b). While in the baseline electrolyte the pouch cells survive only 20 cycles, in which uncontrolled side reactions rapidly deplete the Li and/or electrolyte and cause catastrophic capacity decay (Fig. 6a, c). Adapting the parameters of practical 10-layer pouch cells (Supplementary Table 7)^4^, the cell-level-specific energy of anode-free pouch cells is calculated to be 442.5 Wh kg^−1^, which is highly competitive among the reported LMBs^4,24,60^.

## Discussion

In this work, an anion-enrichment interface is achieved in our designed weakly solvating and dissociated electrolyte by introducing fluorinated linear carboxylic esters to obtain optimal solvating power coupled with the weakly dissociated lithium salts (LiBF_4_ and LiDFOB). The anion-enrichment interface leads to more decomposition of anions in the inner Helmholtz plane and higher reduction potentials of anions, contributing to the inorganic-abundant interfacial chemistry. Accordingly, compact columnar Li deposits are obtained with a high CE of ~98.7% for Li deposition/stripping. The stable cycling of 4.6 V NCM811 and LiCoO_2_ cathode is enabled over 100 cycles with marginal voltage decay, benefiting from the suppressed cathode-electrolyte side reactions, cracking of the NCM secondary particles, and TM dissolution. Consequently, industrial anode-free pouch cells (>200 mAh) are constructed, in which 80% of capacity remains after 100 cycles under harsh testing conditions of high loading mass (4.64 mAh cm^−2^) and lean electrolyte (2.75 g (Ah)^−1^), corresponding to the estimated energy density of 442.5 Wh/kg. Our proposed methodology for anion-enrichment interface in the dilute electrolyte will provide guideline for precise electrolyte engineering to implement high-voltage Ni-rich Li metal batteries.

## Methods

### Electrolyte preparation

The solvents of Ethyl 2-fluoropropionate (98%), ethyl 3,3,3-trifluoropropanoate (98%), ethyl pentafluoropropionate (98%), and fluoroethylene carbonate (FEC, 99%) were purchased from Alfa, Innochem, TCI, and Innochem, respectively. Lithium tetrafluoroborate (LiBF_4_, 99%), Lithium difluoro(oxalato)borate (LiDFOB, 99%), Lithium hexafluorophosphate (LiPF_6_, 99%) were purchased from Acros, Innochem, and Innochem, respectively. All the electrolytes were made and stored in an argon-filled glovebox with O_2_ and H_2_O level <0.1 ppm. 6 mmol LiBF_4_ and 6 mmol LiDFOB were dissolved and stirred in 2 mL tFEP (mFEP or pFEP) and 4 mL FEC to obtain 1 M LiBF_4_ + 1 M LiDFOB in tFEP (mFEP or pFEP)/FEC electrolyte. The 1 M LiPF_6_ EC/DMC electrolyte was purchased from DodoChem.

### Material characterizations

The morphologies of samples were investigated by scanning electron microscopy (Hitachi S-4800) and transmission electron microscopy (JEM 2100Plus, JEOL Limited Corporation, Japan). The surface chemistry of cycled electrodes was analyzed by XPS (ESCALAB 250 Xi, Thermo Fisher) and TOF-SIMS (ION-TOF). All binding energies of XPS were corrected using the signal of carbon at 284.8 eV as an internal standard. Cross-sectioned NCM811 cathodes were prepared using a focused ion beam (IM-40000, Hitachi) and inspected by SEM. For the transition metal (TM) dissolution measurement, coin cells with NMC811 cathode and LMA in different electrolytes were disassembled and washed. Then, inductively coupled plasma atomic emission spectrometry (ICP-AES) (Agilent 7900) was used to determine the TM concentration. The Raman spectra for electrolytes were collected with an NRS-5100 spectrometer (JASCO) using a 532 nm diode-pumped solid-state laser between 4000 and 100 cm^−1^. ^11^B- and ^19^F-NMR spectra were recorded on a Varian Mercury 400-MHz NMR spectrometer at room temperature. The ^23^Na NMR measurements were performed on a Bruker Avance^III^ 800 MHz NMR spectrometer. ^23^Na NMR was carried out in a 10 mM solution of NaTFSI in three solvents (mFEP, tFEP, and pFEP). Note that due to the ultralow permittivity of pFEP, NaTFSI cannot completely dissolve in the pFEP. 0.1 M sodium chloride aqueous solution was used as the reference that was placed in a sealed 1-mm melting point capillary. The capillary was placed in a standard 5-mm NMR tube inserted coaxially into the sample tube. Fourier-transform infrared spectra were measured using a Nicolet iS50 with a diamond-attenuated total reflectance attachment. All the cell disassembly was carried out in an argon-filled glovebox with O_2_ and H_2_O level <0.1 ppm and the electrodes were washed in pure dimethyl ether (DME) (anhydrous, Alfa Aesar, 99.9%) three times to remove the electrolyte, and then the drying samples were obtained and moved to the machine with an argon-filled sealing tube as a transfer box. In this process, all samples would not be exposed to air.

### Electrochemical measurements

Composite cathodes were fabricated by compressing active materials, carbon black (super P), and polyvinylidene fluoride (PVDF) at a weight ratio of 96:2:2. Cell assembly was carried out in an Ar-filled glovebox with O_2_ and H_2_O levels below 0.1 ppm. Coin cells were assembled using Li metal foils with a thickness of 350 μm and diameter of 15.8 mm as the metal electrode and a NCM811 (96% active, mass loading of 22 mg cm^−2^, thickness of 70 μm, and diameter of 12 mm) positive electrode and the polyethylene separator (thickness of 16 μm, diameter of 19 μm, and areal weigh of 1.32 mg cm^−2^). Anode-free pouch cells in this study used a NCM811 positive electrode facing a bare copper current collector as the negative electrode. During the first charge, lithium metal from the positive electrode plates is directly onto the copper current collector. Electrochemical impedance spectroscopy (EIS) was carried out on an Autolab PGSTAT302N (Metrohm, Switzerland) over a frequency range of 1 MHz to 10 mHz. The ionic conductivities of the electrolytes are obtained by measured electrochemical impedance spectroscopy (EIS) using an electrochemical workstation (IM6e Zahner) in the oven at a set temperature. The rate, GITT, and cycling tests for coin cells and pouch cells were carried out on a Land instrument in the oven at 25 °C. For Li stripping/deposition CE, cycling was done by depositing the capacity of 1 mAh cm^−2^ of Li onto the Cu electrode at the current density of 0.5 mA cm^−2^ followed by stripping to 1 V. The average CE was calculated by dividing the total stripping capacity by the total deposition capacity. The Li|NCM811 cells were cycled at C/2 (1C = 200 mA g^−1^) charge/discharge between 3 and 4.6 V after the first three activation cycles at C/5 charge/discharge. The anode-free Cu|NCM811 pouch cells were cycled at 0.1C charge and 0.5 C discharge between 3 and 4.6 V. The specific energy of pouch cells was calculated based on the mass of the cathode, anode, separator, and electrolyte.

### Molecular dynamics (MD) simulation of electrolytes

All the classic molecular dynamic (cMD) simulations conducted in this work were performed using the Large-scale Atomic/Molecular Massively Parallel Simulator (LAMMPS, http://lammps.sandia.gov). All-atom optimized potentials for liquid simulations (OPLS-AA) force-field with the Li^+^, BF_4_^−^, and DFOB^−^ anions description from previous publications^62,91–93^, while the force-field of FEC was obtained from the LigParGen^94^. The electrolyte systems were set up initially with the salt and solvent molecules distributed in the simulation boxes using Packmol^95^ and Moltemplate (http://www.moltemplate.org/)^96^. For each system, an initial energy minimization at 0 K (energy and force tolerances of 10^−5^) was performed to obtain the ground-state structure. After this, the system was heated from 0 K to room temperature (300 K) at constant volume over 0.2 ns using a Langevin thermostat, with a damping parameter of 100 ps. The system was equilibrated in the constant temperature (300 K) and constant pressure (1 bar) (NpT ensemble) for 5 ns. Finally, a MD run in the NVT ensemble was performed for 5 ns for equilibrium, and a following 5 ns NVT simulation was used for analysis. The visualizations were made by using VESTA^97^ and VMD^98^ software.

The classical MD simulations were performed by using LAMMPS at constant volume and temperature (NVT) to the electrolyte-electrode interface with two three-layer Cu electrodes in an FCC lattice sandwiching the 54.22 × 54.22 × 60 Å^3^ electrolyte. A vacuum layer of 50 Å in the z direction is set to ensure no interactions between the periodic electrode sheets in an adjacent cell. A surface charge was applied by placing a partial charge on the first layer of Cu atoms, the surface charge was set to be 0, ±5 µC/cm^2^, and ±10 µC/cm^2^, respectively. Equilibration was performed at a 1 fs time step for 5 ns at 300 K, followed by 10 ns simulation for data collection.

### Quantum chemistry calculations of the solvate reduction potentials

Quantum chemistry calculations were performed using Gaussian 16 software package. Geometry optimizations and energy calculations were performed at the B3LYP/6-311++*G*(d,p) level of theory. The PCM implicit solvent model was used with the ether (*ε* = 4.24) as the implicit solvent. The reduction potential *E*_red_ for the Li solvates denoted as complex A was calculated as the negative of the energy or free energy of formation of A^−^ in solution [Δ*G*_S_ = *G*_S_(A^−^) − *G*_S_(A)] divided by Faraday’s constant as given by Eq. (1):

1 $${{E}}_{{{{{{\rm{red}}}}}}}=-\frac{\varDelta {{G}}_{298{{{{{\rm{k}}}}}}}^{{{{{{\rm{S}}}}}}}}{{F}}-1.23\,{{{{{\rm{V}}}}}}$$

1.23 V converts from the absolute potential to vs. Li.

### Calculation of relative permittivity of solvents

According to the Clausius-Mossotti relation, as shown in Eq. (2)^99^:

2 $$\frac{{\varepsilon }_{{{{{{\rm{r}}}}}}}-1}{{\varepsilon }_{{{{{{\rm{r}}}}}}}+2}=\frac{{N}\alpha }{3{\varepsilon }_{0}}$$

Where *ε*_r_ = *ε*/*ε*_0_ is the relative permittivity, *ε*_0_ is the vacuum permittivity (8.85 × 10^−12^ F/m), *N* is the molecular density (m^−3^), α is the molecular polarizability (C m^2^ V^−1^).

According to the Lorentz-Lorenz formula, as shown in Eqs. (3) and (4)^100,101^:

3 $${{n}}^{2}=1+4\pi \chi$$

4 $$\chi=\frac{{N}\alpha ^{\prime} }{1-(4\pi /3){N}\alpha ^{\prime} }$$

where *n* is the index of refraction, *χ* is the electric susceptibility, *α*′ is the polarizability volume (Å^3^). The molecular polarizability α is often expressed in terms of polarizability volume *α*′ that has volume unit. *α* (1 a.u.) = *α*′ (0.1481847 Å^3^) = 1.6488 × 10^−41^ C^2^ m^2^ J^−1^. Some parameters of solvents can be referred to Supplementary Table 1.

## Supplementary information

Supplementary Information

## Data Availability

Extra data are available from the corresponding author upon reasonable request. Source data are provided with this paper.

## Source data

Source Data
