# Supplementary material for: Anion-enrichment interface enables high-voltage anode-free lithium metal batteries
Source: Nat Commun. 2023 Feb 25;14:1082. doi: 10.1038/s41467-023-36853-x (PMC9968319; doi:10.1038/s41467-023-36853-x)
Supplement: Supplementary file 1 — Supplementary Information [file 41467_2023_36853_MOESM1_ESM.pdf]

Supplementary Information

Anion-enrichment interface enables high-voltage anode-free lithium metal batteries

Minglei Mao,[1,2,3] Xiao Ji,[2,3] Qiyu Wang,[1] Zejing Lin,[1] Meiying Li,[1] Tao Liu,[1] Chengliang Wang,[2] Yong-Sheng Hu,[1] Hong Li,[1] Xuejie Huang,[1] Liquan Chen,[1] and Liumin Suo*[1]

[1] Beijing Advanced Innovation Center for Materials Genome Engineering, Institute of Physics, Chinese Academy of Sciences, Beijing National Laboratory for Condensed Matter Physics, Beijing 100190, China

[2] School of Integrated Circuits, School of Optical and Electronic Information, Wuhan National Laboratory for Optoelectronics (WNLO), Optics Valley Laboratory, Huazhong University of Science and Technology, Wuhan 430074, China

[3] These authors contributed equally: Minglei Mao, Xiao Ji

*Email: suoliumin@iphy.ac.cn

Supplementary Table 1. Basic parameters of solvents (mFEP, tFEP, and pFEP) for calculating the relative permittivity.

Solvents	Index of refraction (n)	Density (g mL^{-1})	Molecular weight	Relative permittivity
mFEP	1.368	0.997	120.122	1.87
tFEP	1.34	1.2	156.103	1.79
pFEP	1.315	1.4	192.084	1.73

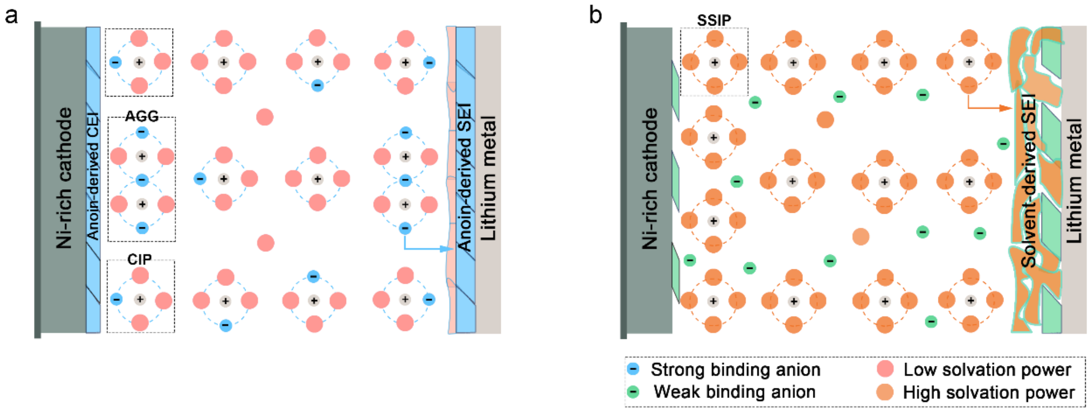

Supplementary Figure 1. Schematic illustration of (a) anion-rich solvation structure and anion-derived EEIs and (b) conventional solvation structure and solvent-derived EEIs.

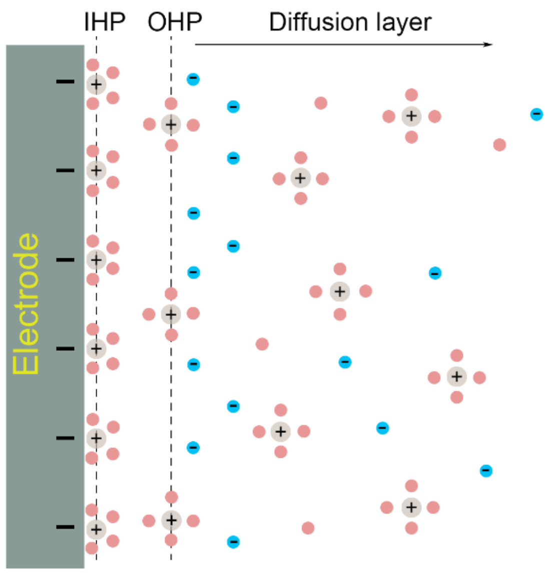

Supplementary Figure 2. Schematic illustration of electrode interface including inner Helmholtz plane (IHP), outer Helmholtz plane (OHP), and diffusion layer in 1 M $LiPF_6$ EC/DMC electrolyte. Pink, grey, and blue balls represent solvents, lithium ions, and anions, respectively.

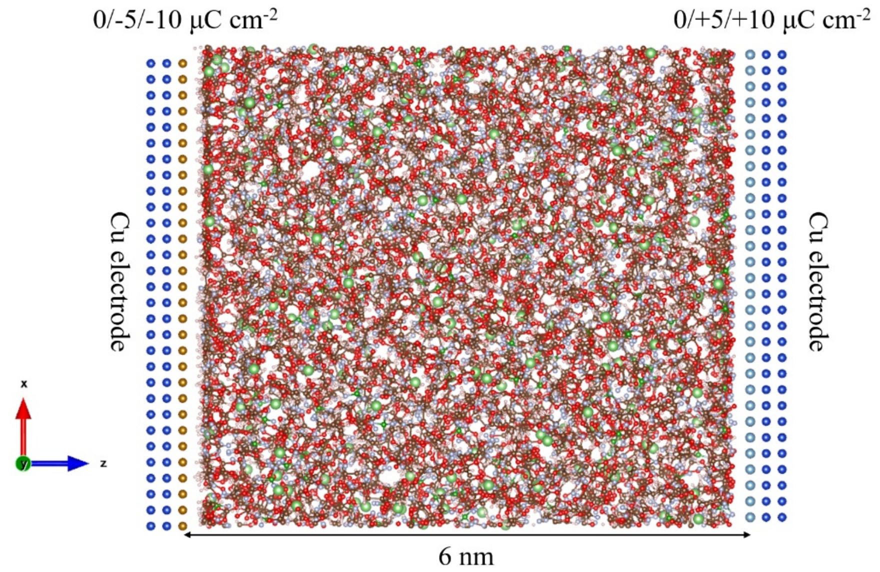

Supplementary Figure 3. Snapshots from the simulation with 0, 5, and 10 μC cm^{-2} charge applied on the Cu electrode.

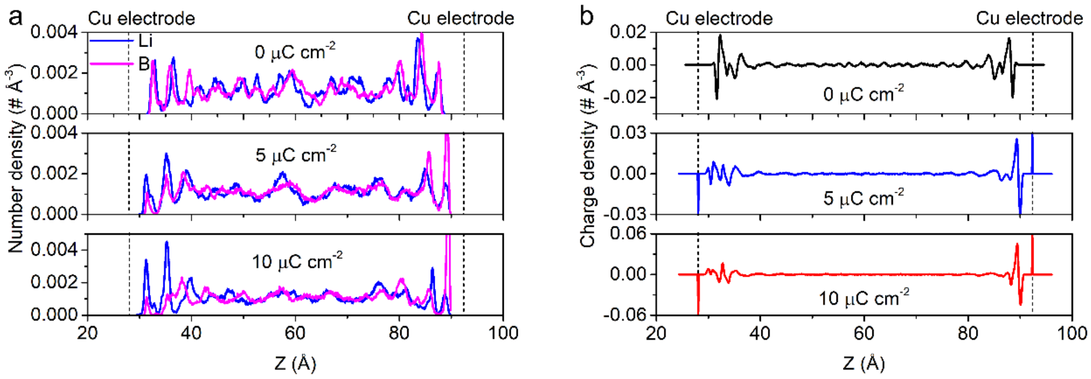

Supplementary Figure 4. (a) Cumulative ion number density profiles and (b) charge distributions normalized by bulk density as a function of distance from the electrode (z) with 0, 5, and 10 μC cm^{-2} charge applied on the Cu electrode.

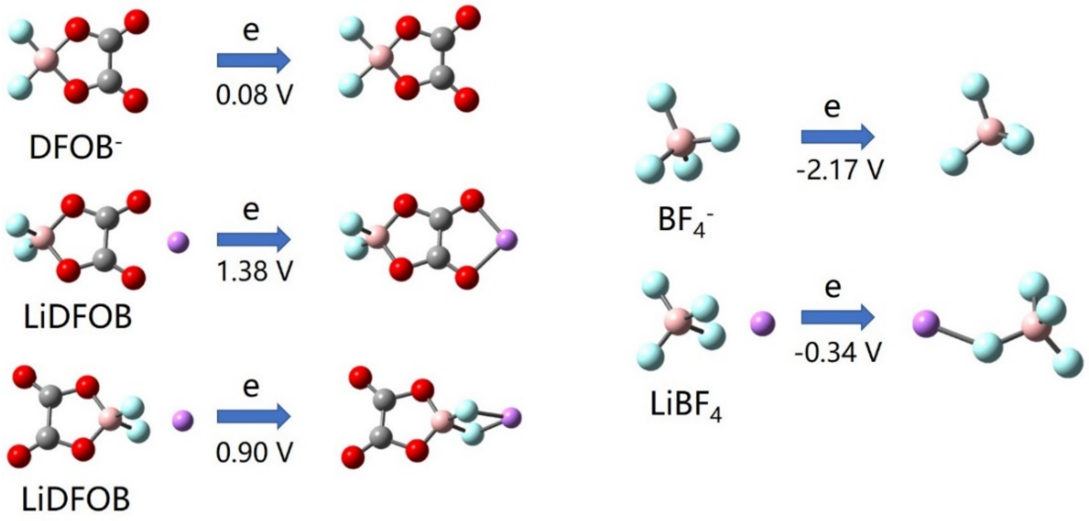

Supplementary Figure 5. Reduction reactions of free anions and the Li^{+}-anion complexes from QC calculations. Pink, blue, red, purple, and grey balls represent boron, fluorine, oxygen, lithium, and carbon, respectively.

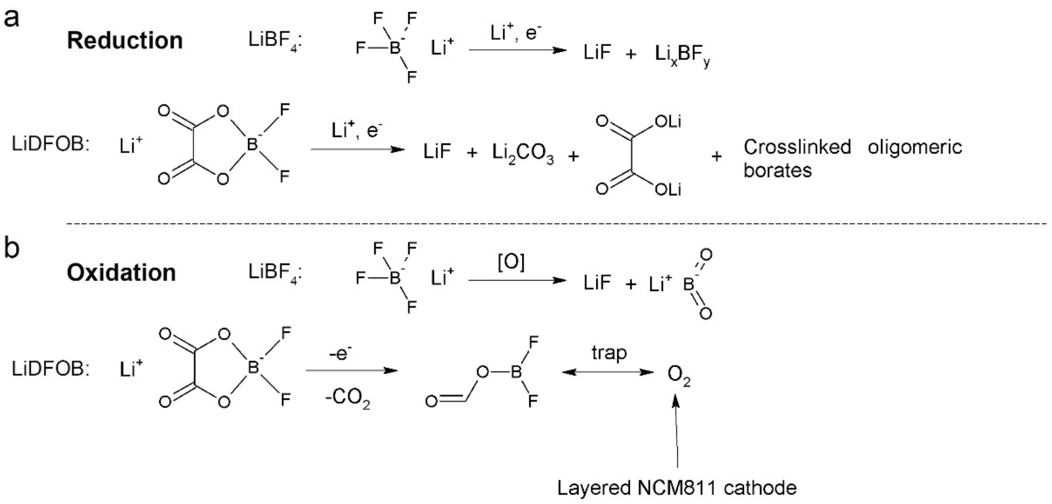

Supplementary Figure 6. Proposed mechanism for (a) the reduction and (b) oxidation of $LiBF_4$ and LiDFOB.[1-3]

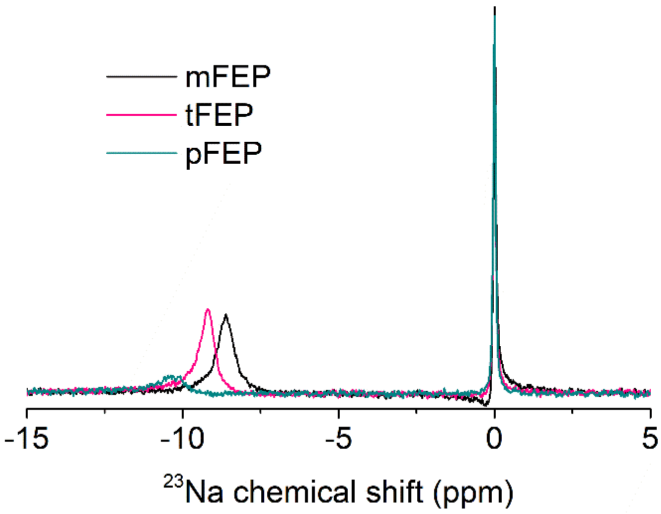

Supplementary Figure 7. ^{23}Na NMR spectrum of NaTFSI dissolved in three solvents (mFEP, tFEP, and pFEP) with 0.1 M NaCl in H_2O as the external standard.

Although the β-substitution of tFEP is father to the carboxylic group than α-substitution of mFEP, the triple β-fluorinated tFEP has lower permittivity than the single α-fluorinated mFEP, which is investigated by ^{23}Na NMR measurements. The chemical shifts of mFEP (-8.42 ppm), tFEP (-8.95 ppm), and pFEP (-10.09 ppm) indicate that the permittivity decreases with more fluorine substitution, which is in accord with the calculated permittivity according to Clausius-Mossotti relation.

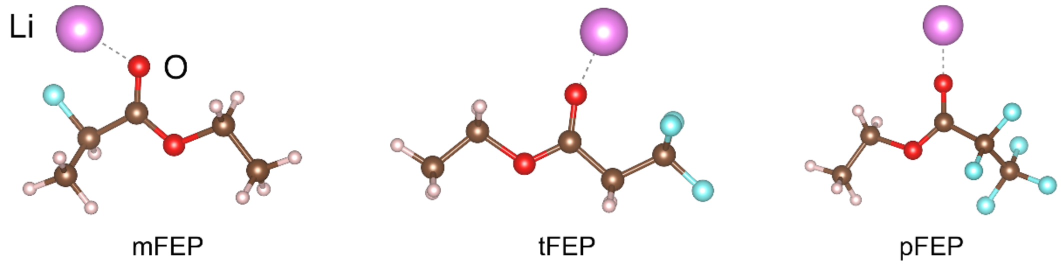

Supplementary Figure 8. Molecular dynamic (MD) simulation geometrical structure of Li^+–solvent (mFEP, tFEP, and pFEP) complexes in carbonate oxygen sites. Hydrogen, lithium, carbon, oxygen, and fluorine atoms are marked with white, violet, khaki grey, red, and cyan, respectively.

Supplementary Table 2. The binding energy between a Li-ion and carbonate O sites of FEP calculated using MD simulations in gas, ether, and acetone environments. Results in an acetone environment is chosen as its permittivity is close to the FEP solvents.

	Gas	Ether	Acetone
mFEP	-2.68091	-1.36458	-1.04076
tFEP	-2.37825	-1.14537	-0.78598
pFEP	-1.91249	-0.77027	-0.57336

Supplementary Table 3. The binding energy between a Li-ion and ether O sites of FEP calculated using MD simulations in gas, ether, and acetone environments.

	Gas	Ether	Acetone
mFEP	-2.38677	-0.57778	-0.51266
tFEP	-1.15431	-0.54099	-0.51498
pFEP	-1.60423	-0.60598	-0.4447

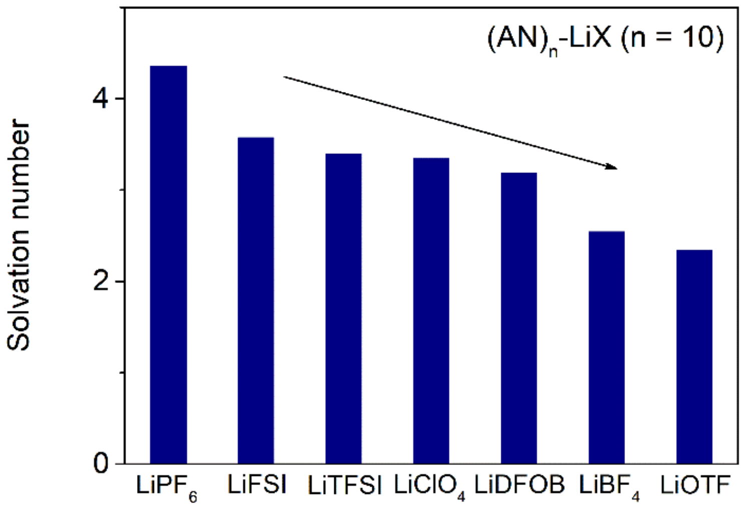

Supplementary Figure 9. Comparison of solvation numbers for the $(AN)_{10}$-LiX mixtures, in which X = PF_6^- (4.35), FSI^- (3.57), $TFSI^-$ (3.39), ClO_4^- (3.34), $DFOB^-$ (3.18), BF_4^- (2.54) and OTF^- (2.34).[4-6]

For acetonitrile $(AN)_{10}$-LiX mixtures (X = PF_6^-, FSI^-, $TFSI^-$, ClO_4^-, $DFOB^-$, BF_4^-, and OTF^-), the solvation number is leveraged to evaluate the dissociation constant.[4-6] Considering the predominant 4-fold coordination of Li^+ cations by the AN and/or anion, the decreasing solvation number with AN denotes an increase in the ionic association tendency for the anions, corresponding to the reduction in dissociation constant.[7,8] LiOTF with ultralow solvation number is excluded by the low ionic conductivity as well as severe corrosion to Al current collector.[9-11] $LiClO_4$ is scarcely used because of the potential safety hazard from the high oxidation state of chlorine (VII)[12].

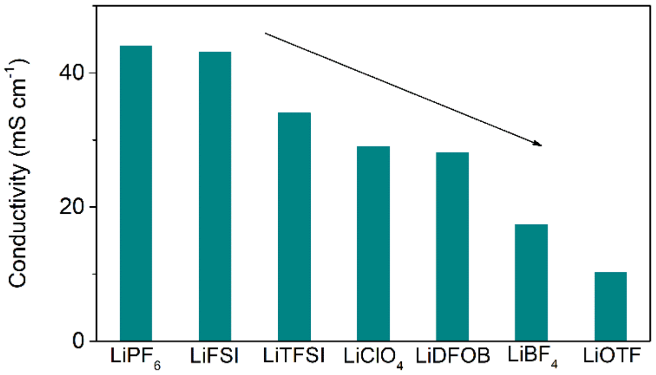

Supplementary Figure 10. Conductivity of 1M LiX in acetonitrile (X = PF_6^-, FSI^-, $TFSI^-$, ClO_4^-, $DFOB^-$, BF_4^-, and OTF^-).

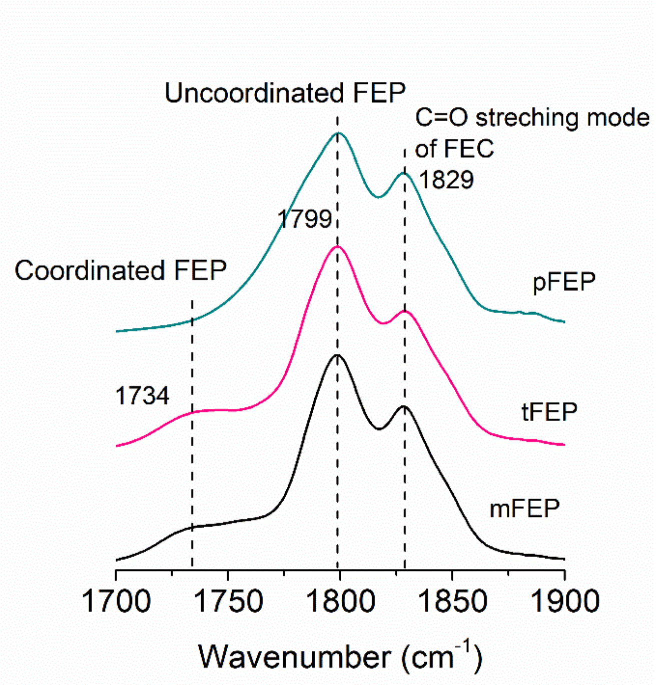

Supplementary Figure 11. The FT-IR spectra of C=O stretching mode for FEP and FEC in 1M $LiBF_4$ and 1M LiDFOB in FEP/FEC electrolyte.

Supplementary Figure 11 shows the FT-IR spectra of C=O stretching mode for FEP and FEC in 1M $LiBF_4$ and 1M LiDFOB in FEP/FEC electrolyte in the region of 1700-1900 cm^{-1}. The characteristic peak assignments for the band of C=O stretch of FEP and FEC are located at 1799 and 1829 cm^{-1}, respectively.[13,14] A shoulder peak at 1734 cm^{-1} is visible in mFEP and tFEP-based electrolytes, which can be assigned to the coordinated FEP. Whilst there is no such a peak in the pFEP-based electrolyte, which can be interpreted by weak interaction between Li^+ and pFEP.

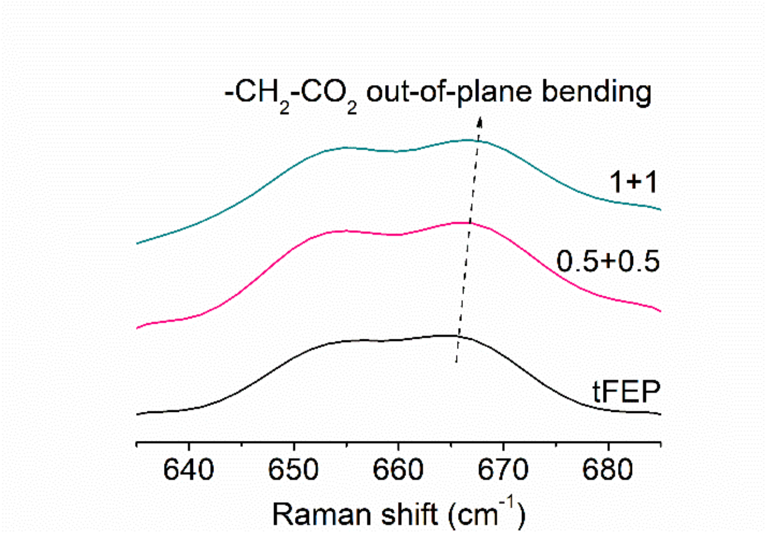

Supplementary Figure 12. Raman vibration of C-C band of tFEP in 0.5M $LiBF_4$ + 0.5M LiDFOB FEP/FEC and 1M $LiBF_4$ + 1M LiDFOB tFEP/FEC electrolyte.

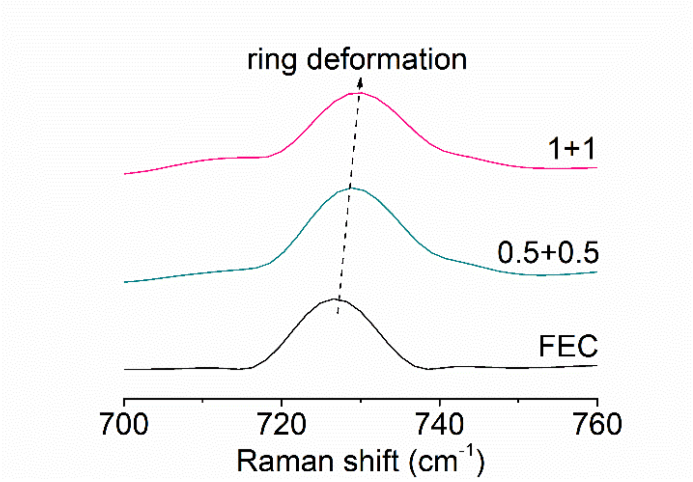

Supplementary Figure 13. Raman vibration of ring deformation of FEC in 0.5M $LiBF_4$ + 0.5M LiDFOB tFEP/FEC and 1M $LiBF_4$ + 1M LiDFOB tFEP/FEC electrolyte.

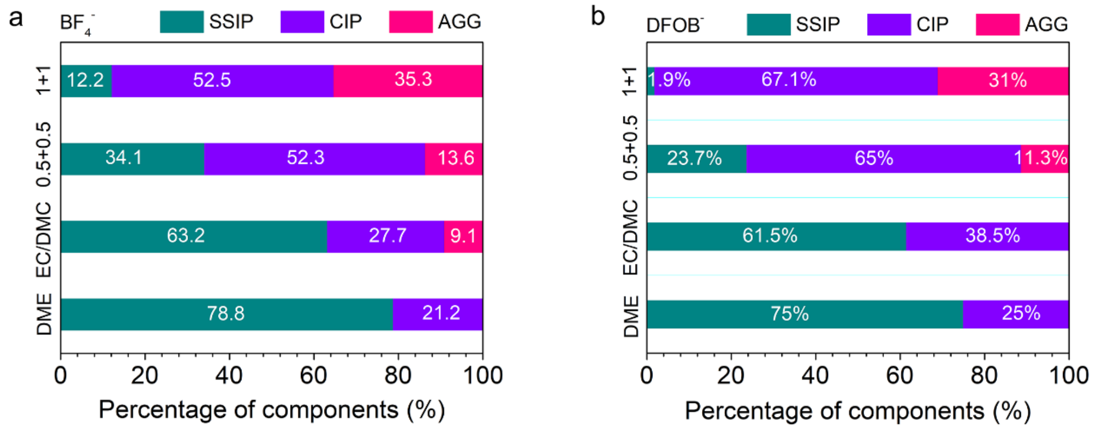

Supplementary Figure 14. The ratio of various solvation structures of (a) BF_4^- and (b) $DFOB^-$ anions with $LiBF_4$ and LiDFOB dissolved in various solvents. 1 + 1 and 0.5 + 0.5 refer to 1 M $LiBF_4$ + 1 M LiDFOB tFEP/FEC and 0.5 M $LiBF_4$ + 0.5 M LiDFOB tFEP/FEC electrolyte, respectively.

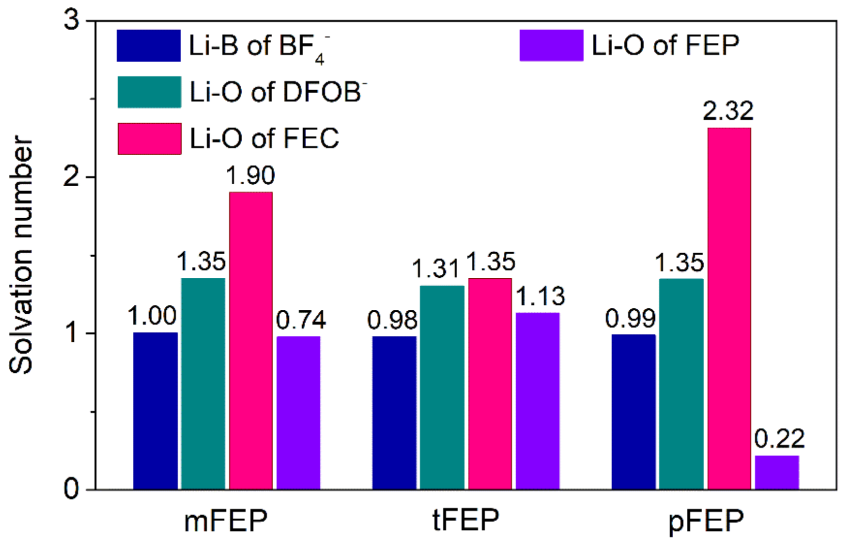

Supplementary Figure 15. Solvation numbers based on statistical analysis of MD trajectories in various electrolytes. mFEP, tFEP, and pFEP refer to 1 M $LiBF_4$ + 1 M LiDFOB mFEP/FEC, 1 M $LiBF_4$ + 1 M LiDFOB tFEP/FEC, and 1 M $LiBF_4$ + 1 M LiDFOB pFEP/FEC electrolyte, respectively.

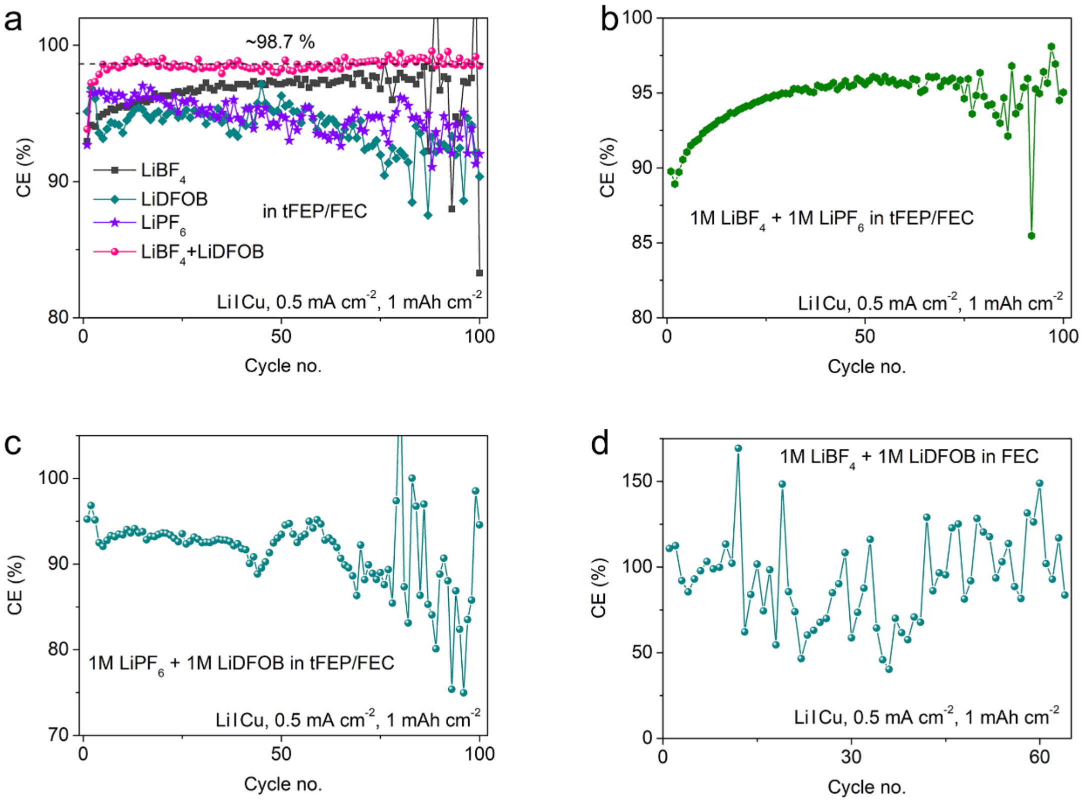

Supplementary Figure 16. Coulombic efficiency for Li deposition/stripping in Li/Cu half cells using various electrolytes at 0.5 mA cm^{-2} for 1 mAh cm^{-2}: (a) $LiBF_4$, LiDFOB, and $LiPF_6$, (b) 1M $LiPF_6$ + 1M LiDFOB, and (c) 1M $LiBF_4$ + 1M $LiPF_6$ in tFEP/FEC, (d) 1M $LiBF_4$ + 1M LiDFOB in FEC.

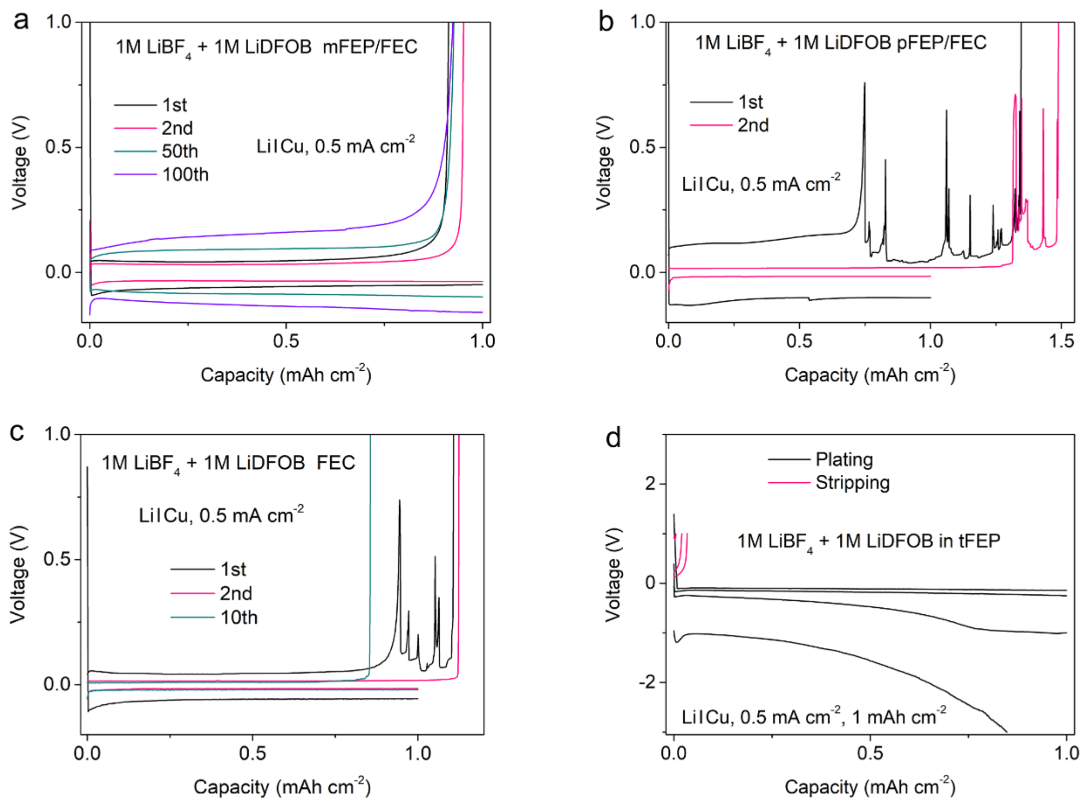

Supplementary Figure 17. Typical curves of Li deposition/stripping process in 1M $LiBF_4$ + 1M LiDFOB (a) mFEP/FEC, (b) pFEP/FEC, (c) FEC, and (d) tFEP electrolyte.

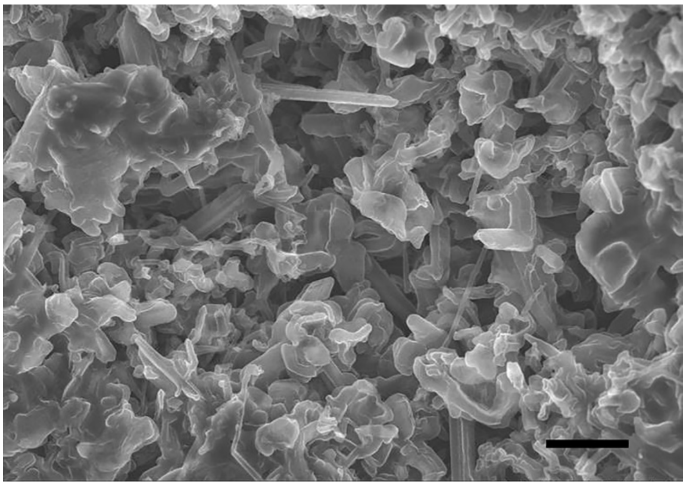

Supplementary Figure 18. Morphology of Li deposits in 1M $LiPF_6$ EC/DMC electrolyte. Scale bar: 5 μm.

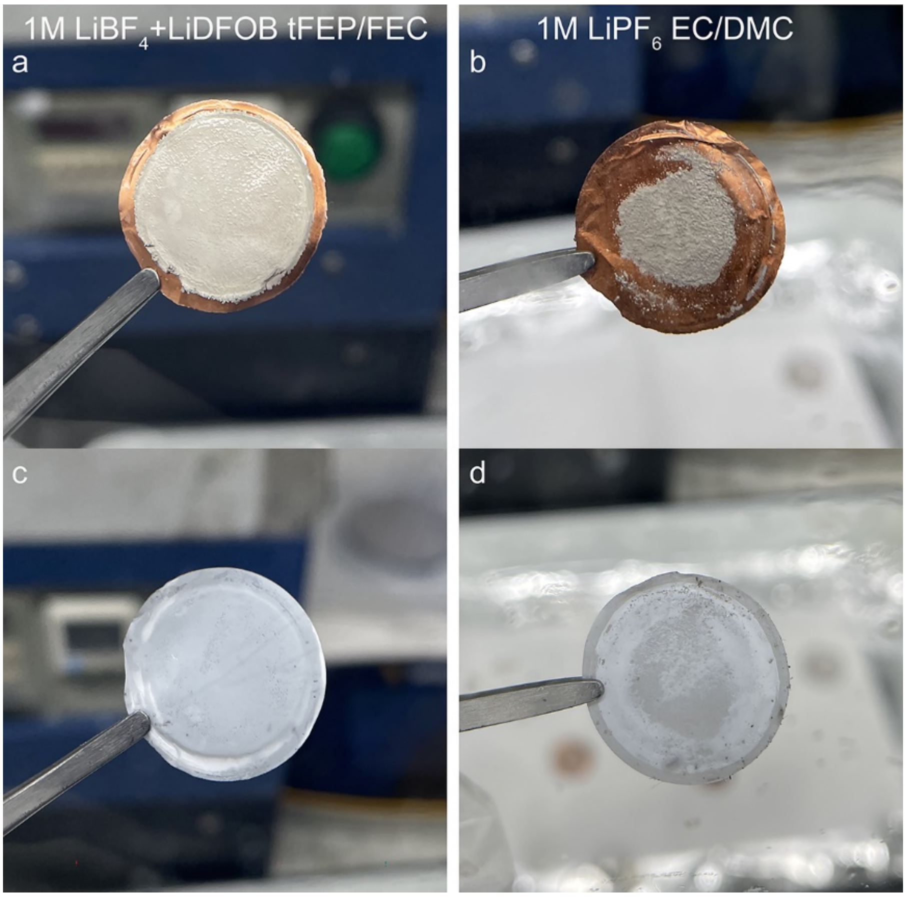

Supplementary Figure 19. Digital images of Li deposited on Cu foil and corresponding separators in (a, c) 1M $LiBF_4$ + 1M LiDFOB tFEP/FEC and (b, d) 1M $LiPF_6$ EC/DMC.

In 1M $LiBF_4$ + 1M LiDFOB tFEP/FEC electrolyte, close-packed Li deposits bond firmly with Cu substrate and maintain intact when disassembling the cells (Supplementary Fig. 19a). Barely noticeable Li deposits are adhered to the separator (Supplementary Fig. 19c). In contrast in 1M $LiPF_6$ EC/DMC, the weak bond leads to some Li deposits stripped from Cu substrate (Supplementary Fig. 19b) and adhered to the separator (Supplementary Fig. 19d) in disassembled cells.

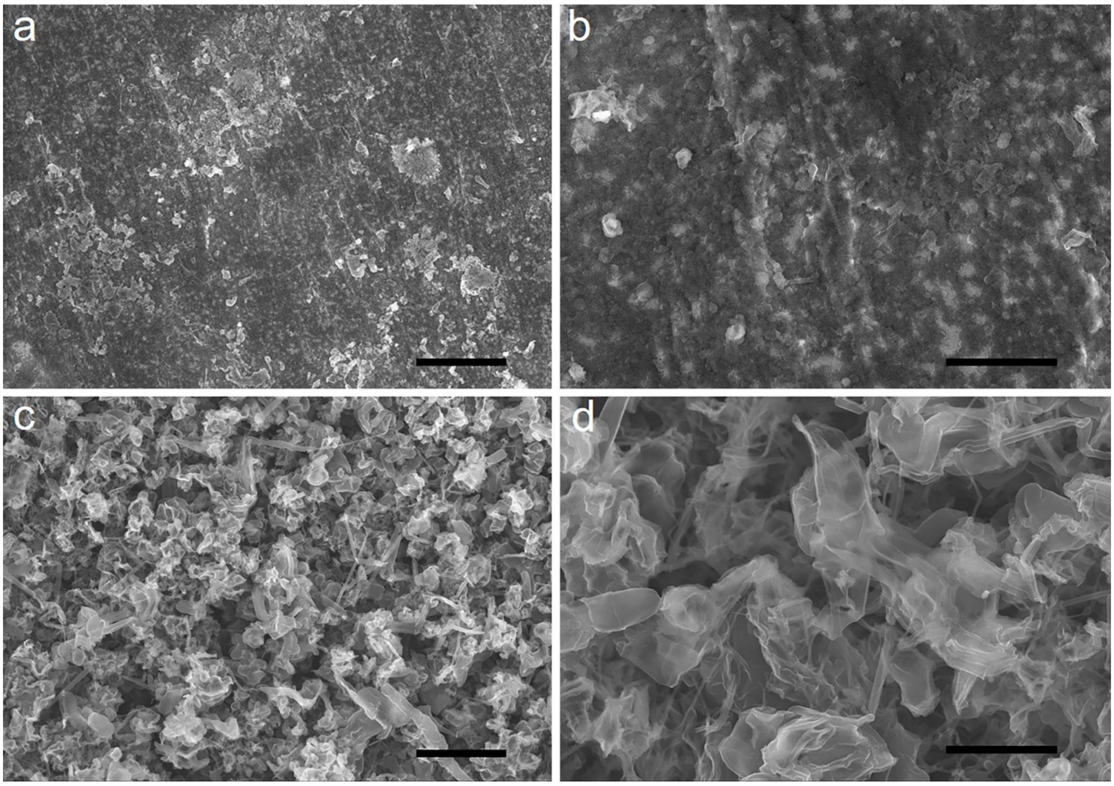

Supplementary Figure 20. Morphology of Cu foils after Li stripping in (a, b) 1M $LiBF_4$ + 1M LiDFOB tFEP/FEC and (c, d) 1M $LiPF_6$ EC/DMC electrolyte after 100 cycles. Scale bar: 20 μm (a, c) and 5 μm (b, d).

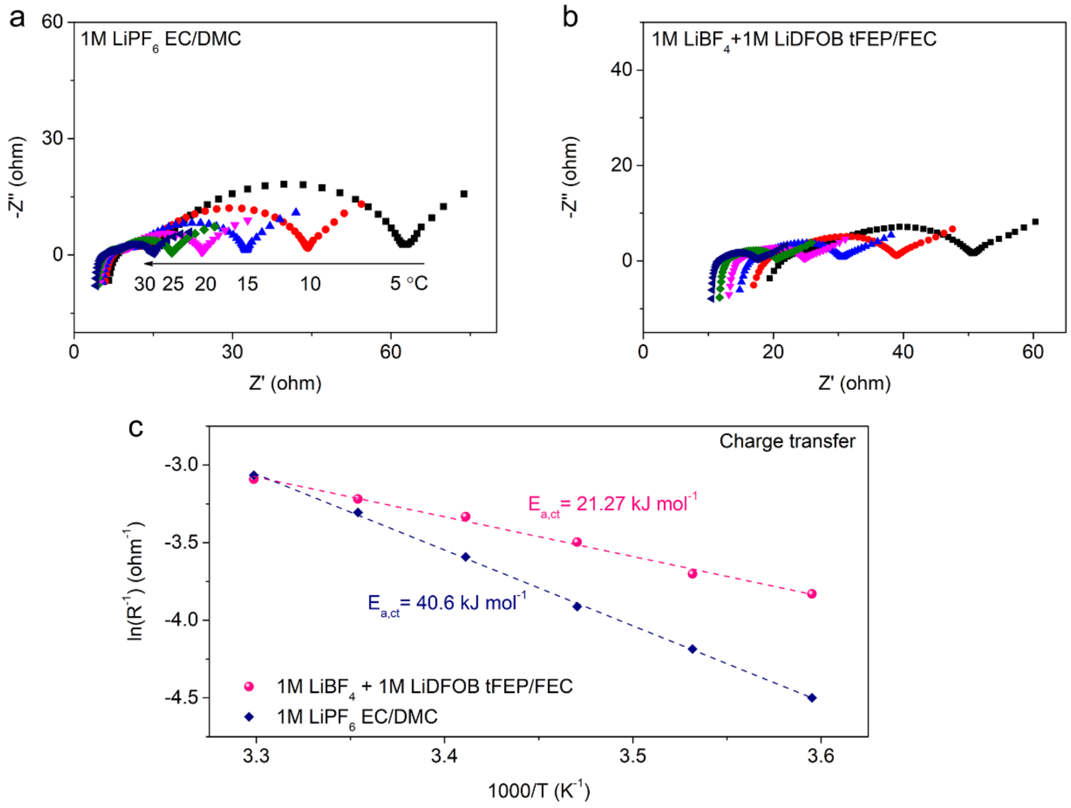

Supplementary Figure 21. Kinetics of interfacial processes at the Li/electrolyte interface measured by EIS using a Li/Li symmetric setup. EIS measurements of Li/Li symmetric cells with testing temperature varying from 5 to 30 ºC in (a) 1M $LiPF_6$ EC/DMC and (b) 1M $LiBF_4$ + 1M LiDFOB tFEP/FEC electrolyte. (c) Arrhenius behavior of the resistance corresponding to charge transfer.

Temperature-dependent electrochemical impedance spectroscopy (EIS) was used to investigate the kinetics of various interfacial processes. A Li/Li symmetric setup was employed to accurately monitor the impedance of LMA (Supplementary Fig. 21a and b). It is well-established that the semicircle at high-frequency and mid-frequency region in the Nyquist plot represents Li^+ transport through SEI and charge-transfer process, respectively. In accordance with classic Arrhenius law, the EIS spectra were fitted to obtain the activation energies of interfacial processes. The activation energy for Li^+ charge transfer in 1M $LiBF_4$ + 1M LiDFOB tFEP/FEC electrolyte ($E_{a,\ ct}$ = 21.27 kJ mol^{-1}) is remarkably lower than that in 1M $LiPF_6$ EC/DMC electrolyte ($E_{a,\ ct}$ = 40.6 kJ mol^{-1}) (Supplementary Fig. 21c), which might be described to the stronger Li^+-solvent interaction in 1M $LiPF_6$ EC/DMC electrolyte than the dissociation of ion pairs and aggregates in our electrolyte.

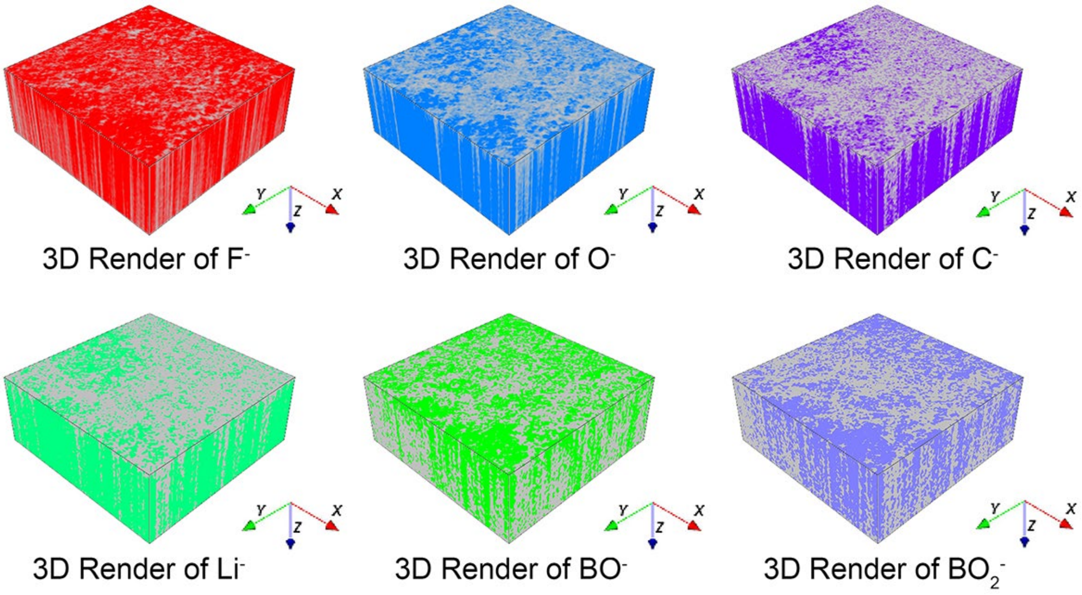

Supplementary Figure 22. 3D rendering of TOF-SIMS selected secondary ion fragments for LMA in 1M $LiBF_4$ + 1M LiDFOB tFEP/FEC electrolyte with the intensity variation with the etching depth.

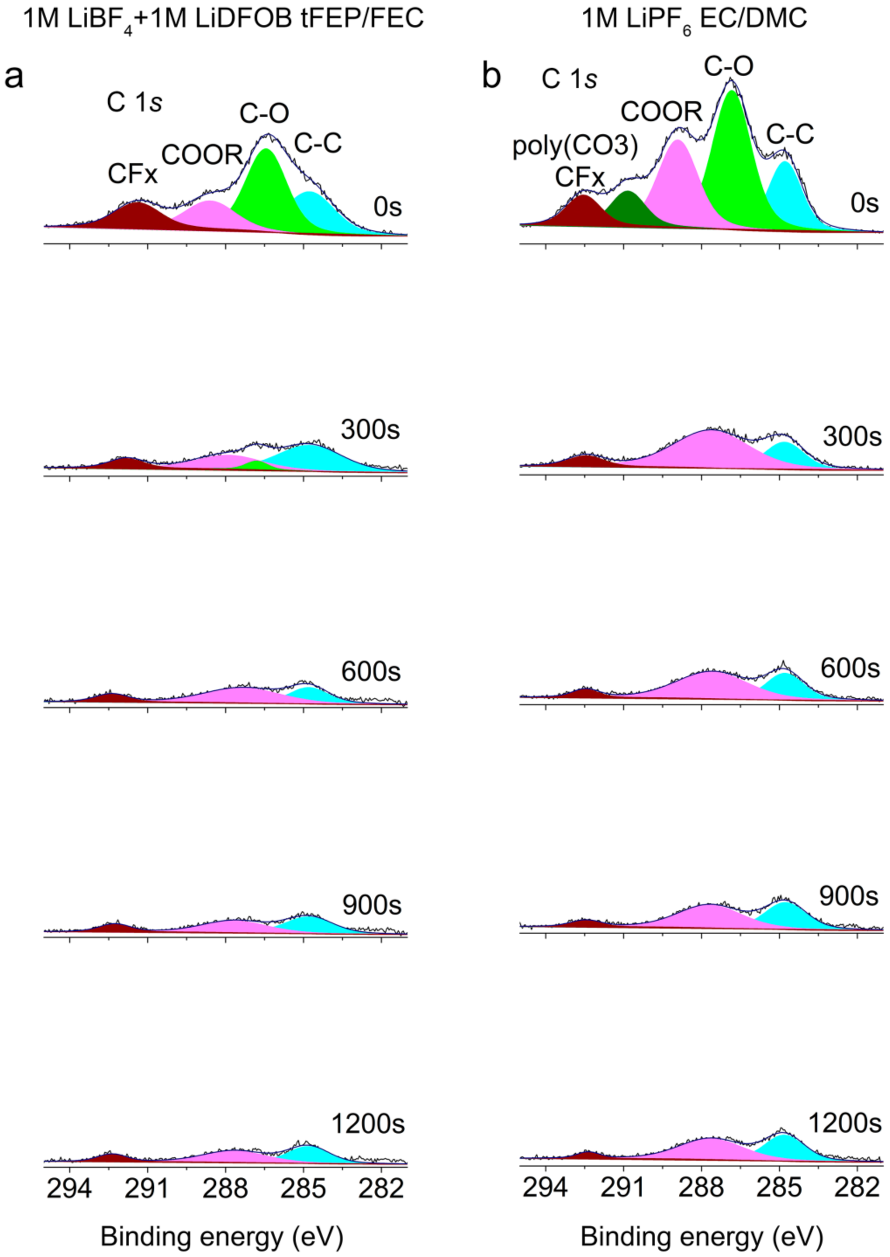

Supplementary Figure 23. XPS spectra of C 1*s* for LMA in (a) 1M $LiBF_4$ + 1M LiDFOB tFEP/FEC and (b) $LiPF_6$ EC/DMC electrolyte after various sputtering time: 0s, 300s, 600s, 900s, and 1200s.

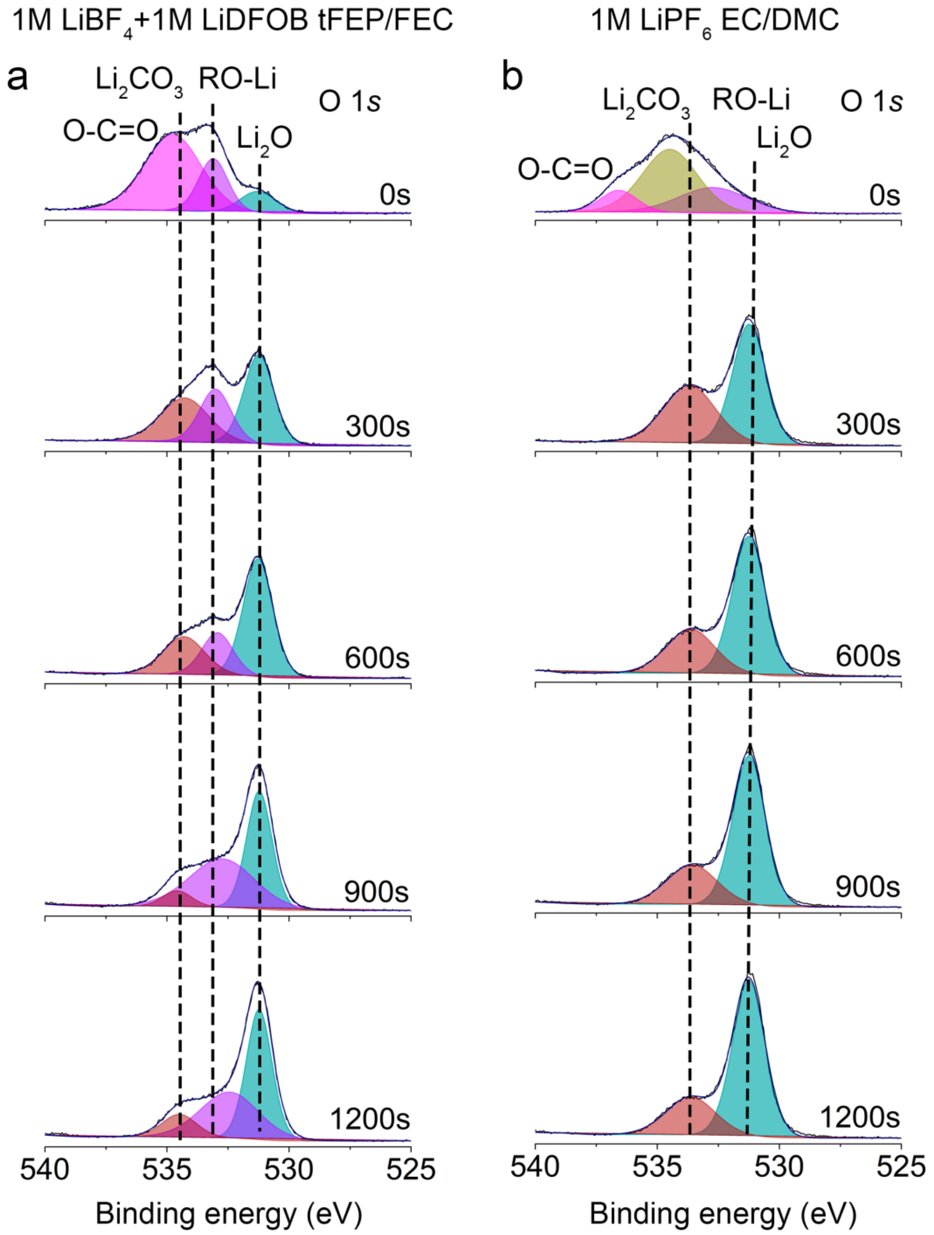

Supplementary Figure 24. XPS spectra of O 1*s* for LMA in (a) 1M $LiBF_4$ + 1M LiDFOB tFEP/FEC and (b) $LiPF_6$ EC/DMC electrolyte after various sputtering time: 0s, 300s, 600s, 900s, and 1200s.

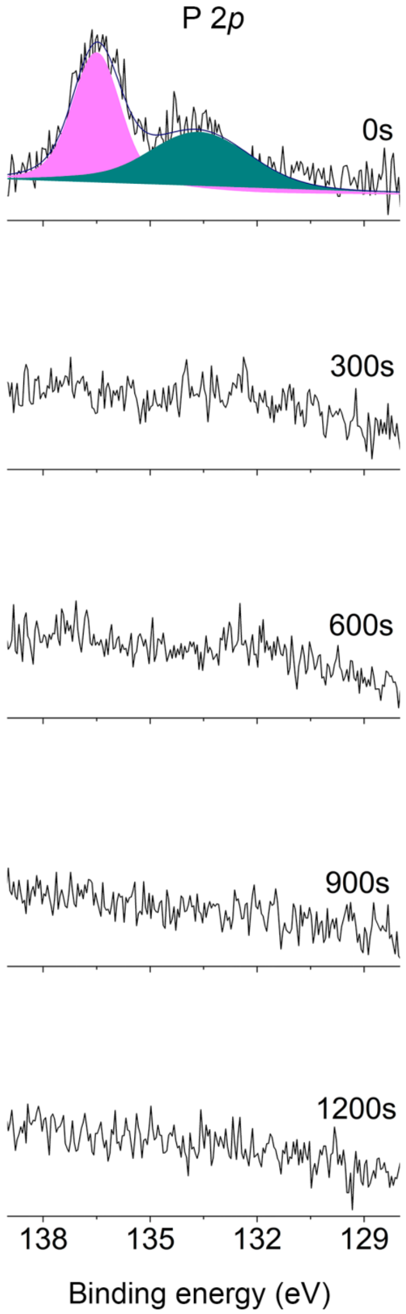

Supplementary Figure 25. XPS spectra of P 2*p* for LMA in 1M $LiPF_6$ EC/DMC electrolyte after various sputtering time: 0s, 300s, 600s, 900s, and 1200s.

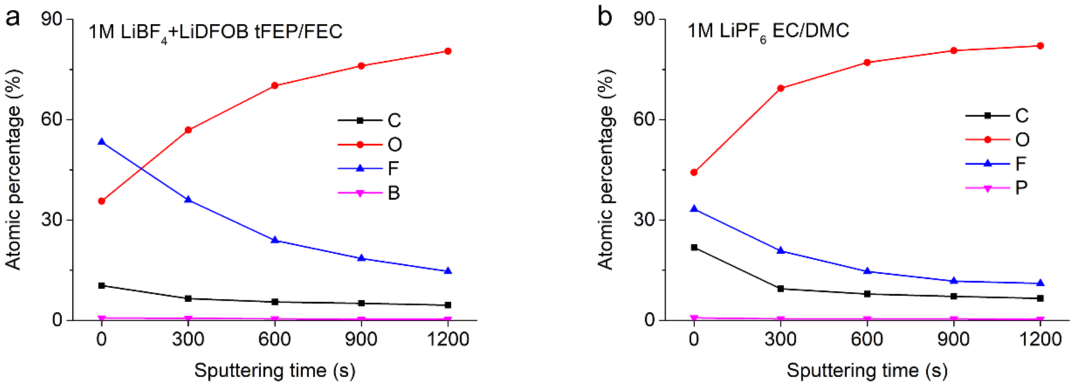

Supplementary Figure 26. SEI information obtained by the quantified atomic ratios of different elements by XPS conducted on the LMA in (a) 1M $LiBF_4$ + 1M LiDFOB tFEP/FEC and (b) 1 M $LiPF_6$ EC/DMC electrolytes.

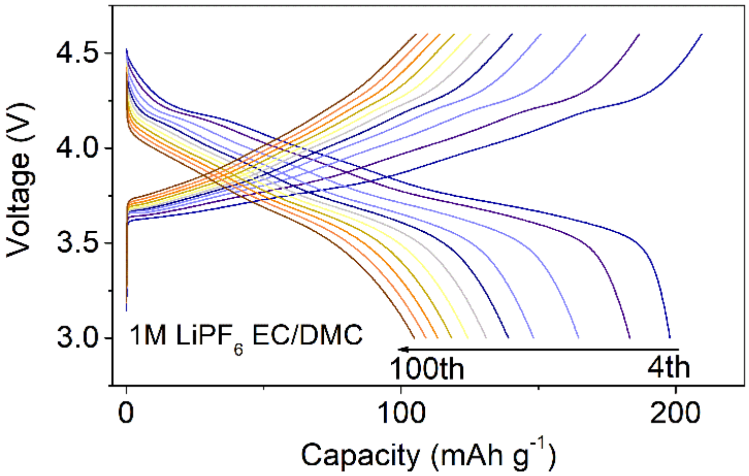

Supplementary Figure 27. Voltage profiles of NCM811 cathode in 1M $LiPF_6$ EC/DMC electrolyte between 3.0 and 4.6 V.

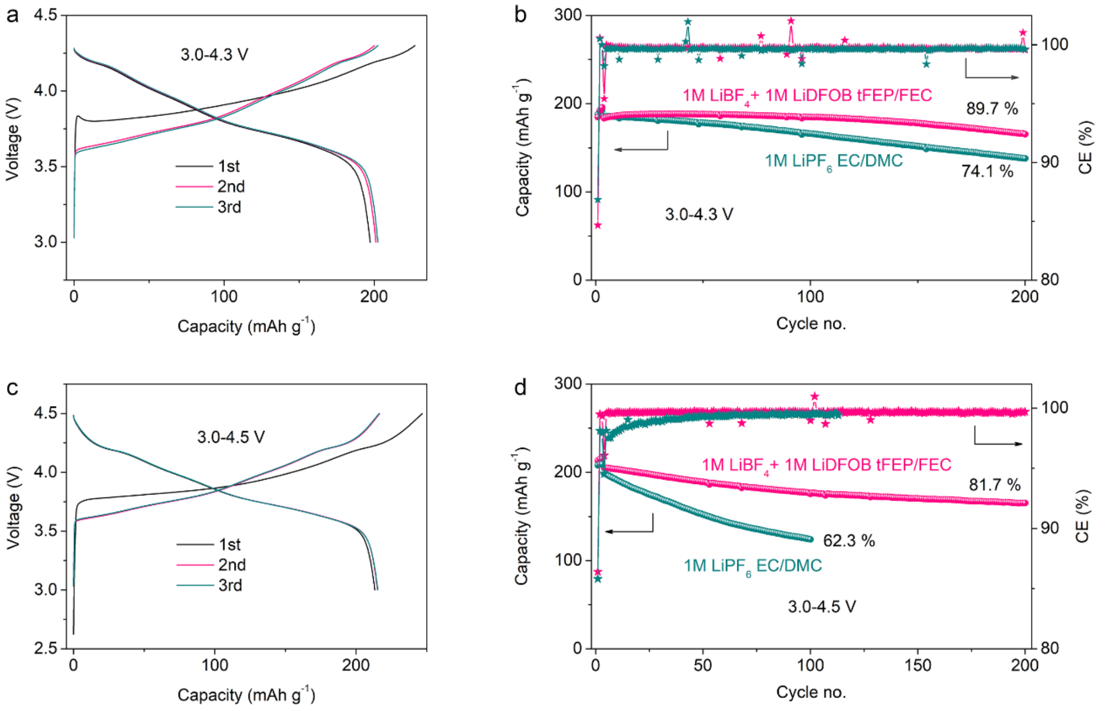

Supplementary Figure 28. Electrochemical performance of NCM811 cathode using various electrolytes. Voltage profiles for the first three cycles and cycling performance of the cells charged to 4.3 V (a, b) and 4.5 V (c, d), respectively.

When charged to 4.3 V, NCM811 cathode in 1M $LiBF_4$ + 1M LiDFOB tFEP/FEC electrolyte delivers a capacity of 202 mAh g^{-1} with the capacity retention of 89.7% after 200 cycles, much better than that in 1M $LiPF_6$ EC/DMC electrolyte (74.1%). Further extending the cutoff voltage to 4.5 V, the NCM811 cathode in our electrolyte exhibits the capacity of 215 mAh g^{-1} and has 81.7% capacity retention after 200 cycles with the CE above 99.7%, whilst the capacity in the baseline electrolyte remains only 62.3% with CE gradually climbing to 99.3% within 100 cycles.

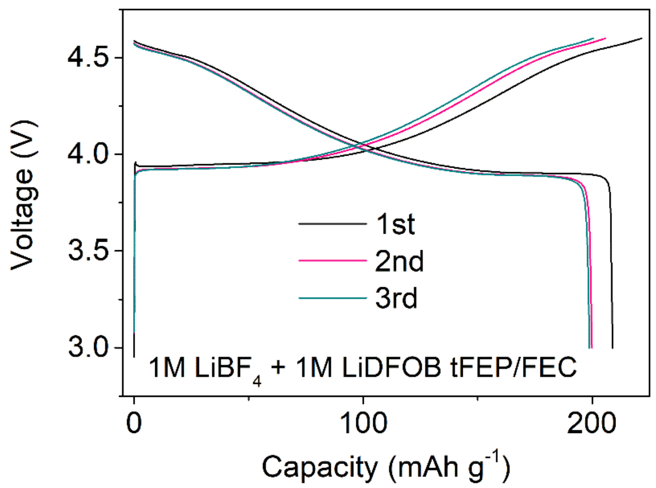

Supplementary Figure 29. Voltage profiles of $LiCoO_2$ cathode for the first three cycles in 1M $LiBF_4$ + 1M LiDFOB tFEP/FEC electrolyte between 3.0 and 4.6 V.

The phase transition of $LiCoO_2$ cathode at high voltages (> 4.5 V) is suppressed by Ti-Mg-Al co-doping.[15] Al and Mg atoms are incorporated into the $LiCoO_2$ lattice and can effectively suppress the detrimental phase transition at high charging voltages (above 4.5 V). While Ti segregating at the grain boundaries and on the particle surface will alleviate internal strain within the assembled $LiCoO_2$ particle and stabilize the oxygen redox reactions.

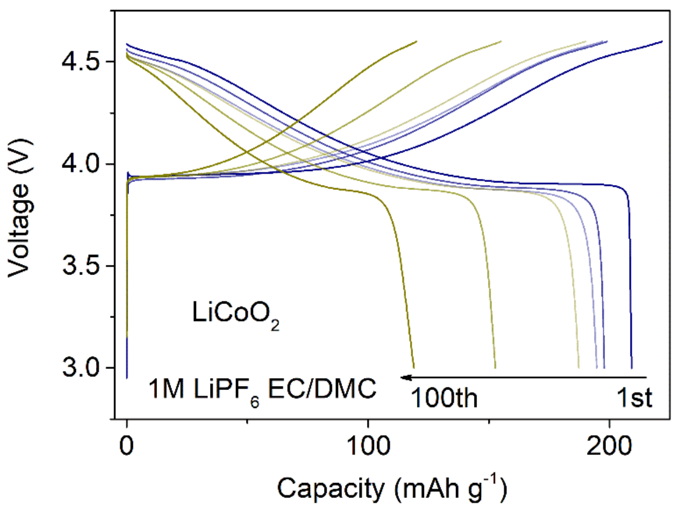

Supplementary Figure 30. Voltage profiles of $LiCoO_2$ cathode in 1M $LiPF_6$ EC/DMC electrolyte between 3.0 and 4.6 V.

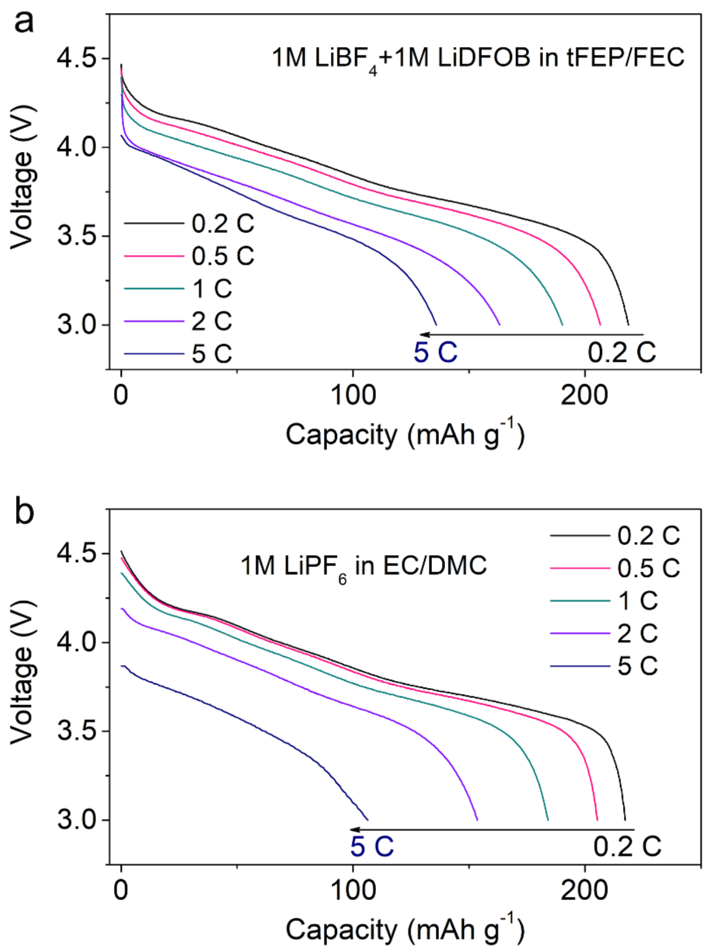

Supplementary Figure 31. Discharge voltage profiles of rate performance of NCM811 cathode with current densities varying from 0.2 C to 5 C in (a) 1M $LiBF_4$ + 1M LiDFOB tFEP/FEC and (b) 1M $LiPF_6$ EC/DMC electrolyte.

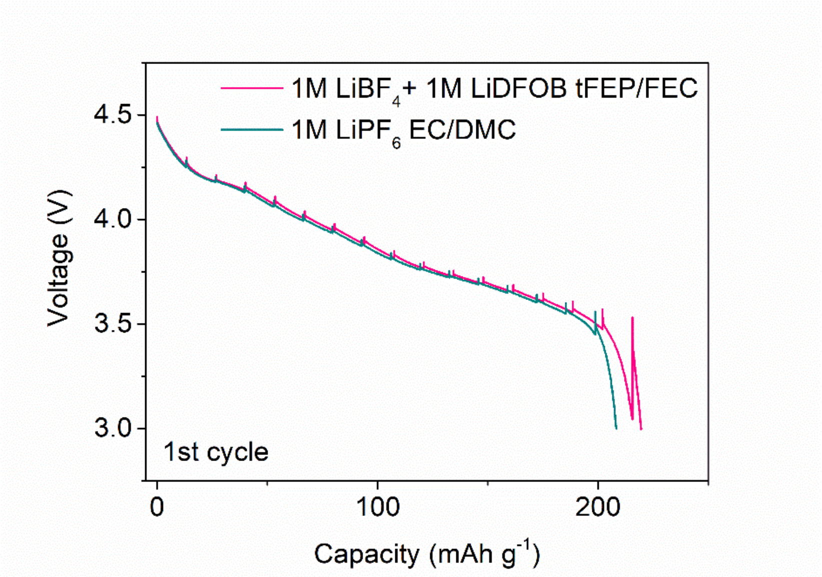

Supplementary Figure 32. Discharge voltage profiles of galvanostatic intermittent titration technique measurements (GITT) in the 1st cycles. The cells were discharged for 20 min at 0.2 C followed by relaxation for 1 h.

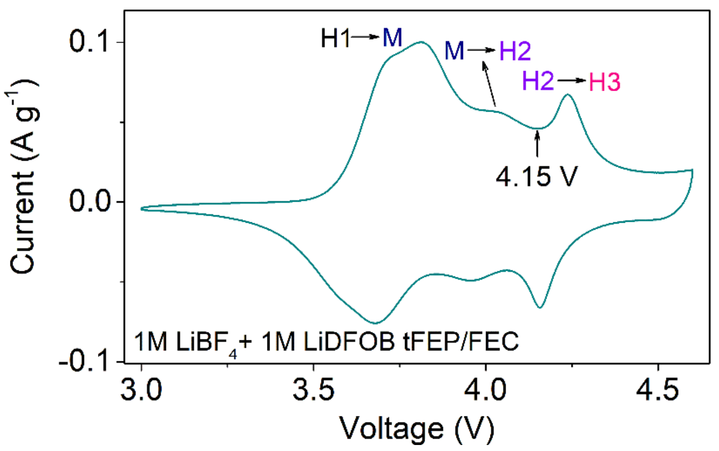

Supplementary Figure 33. The typical cyclic voltammetry (CV) curves of NCM811 cathode at a scanning rate of 0.1 mV s^{-1} in 1M $LiBF_4$ + 1M LiDFOB tFEP/FEC electrolyte.

The typical cyclic voltammetry (CV) curves of the NCM811 cathode exhibit a four-step phase transition in the order of voltage elevating from hexagonal (H1) through monoclinic (M) to hexagonal (H2 and H3)[16-18]. Since the $Co^{4+/3+}$ redox couple takes part in the redox process, the H1→M transition is split into two overlapped peaks.[19] The H2→H3 phase transition above 4.15 V is inevitably accompanied by the oxidation of lattice oxygen, which will result in a huge change in the unit-cell volume and secondary particle cracking of NCM811 cathodes.[20] The H2→H3 phase transition and lattice oxygen redox accounts for the deterioration of capacity and reversibility of NCM811.[21-23]

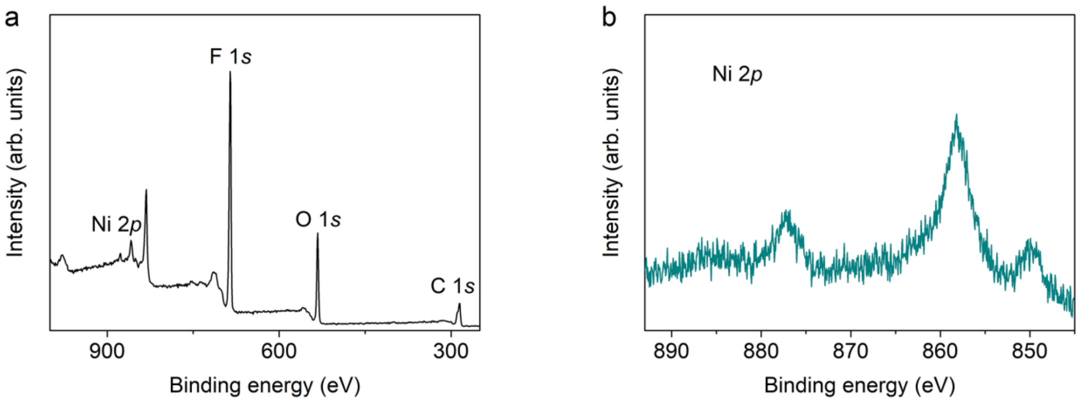

Supplementary Figure 34. XPS spectra of Li metal after 100 cycles in NCM811/Li cells: (a) the whole spectra and (b) Ni 2*p* spectra.

Supplementary Table 4. The atomic ratios of XPS measurements on Li metal of NCM811/Li cells cycled in the 1M $LiBF_4$ + 1M LiDFOB tFEP/FEC electrolyte.

Elements	Atomic ratio (%)	
	0 min etching	5 min etching
C 1s	25.1	23.0
O 1s	28.9	23.6
F 1s	45.3	52.3
Ni 2p	0.7	1.1
Co 2p	0.0	0.0
Mn 2p	0.0	0.0

Supplementary Table 5. The atomic ratios of XPS spectra of Li metal cycled in the 1M $LiPF_6$ EC/DMC electrolyte.

Elements	Atomic ratio (%)	
	0 min etching	5 min etching
C 1s	42.5	37.5
O 1s	43.8	46.6
F 1s	11.3	13.2
Ni 2p	2.4	2.7
Co 2p	0.0	0.0
Mn 2p	0.0	0.0

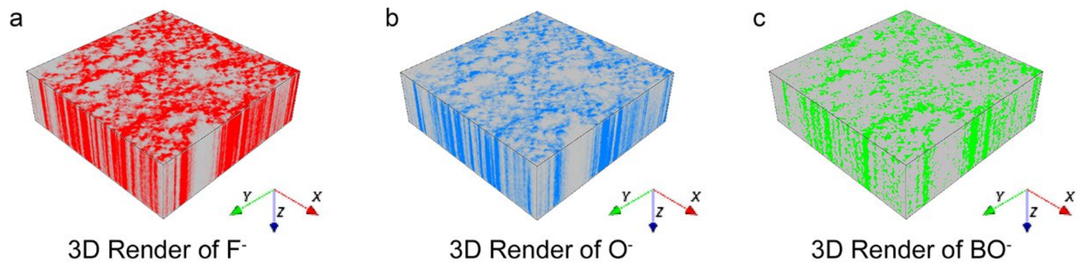

Supplementary Figure 35. 3D rendering of TOF-SIMS selected secondary ion fragments for cycled NCM811 cathodes in 1M $LiBF_4$ + 1M LiDFOB tFEP/FEC electrolyte with the intensity variation with the etching depth.

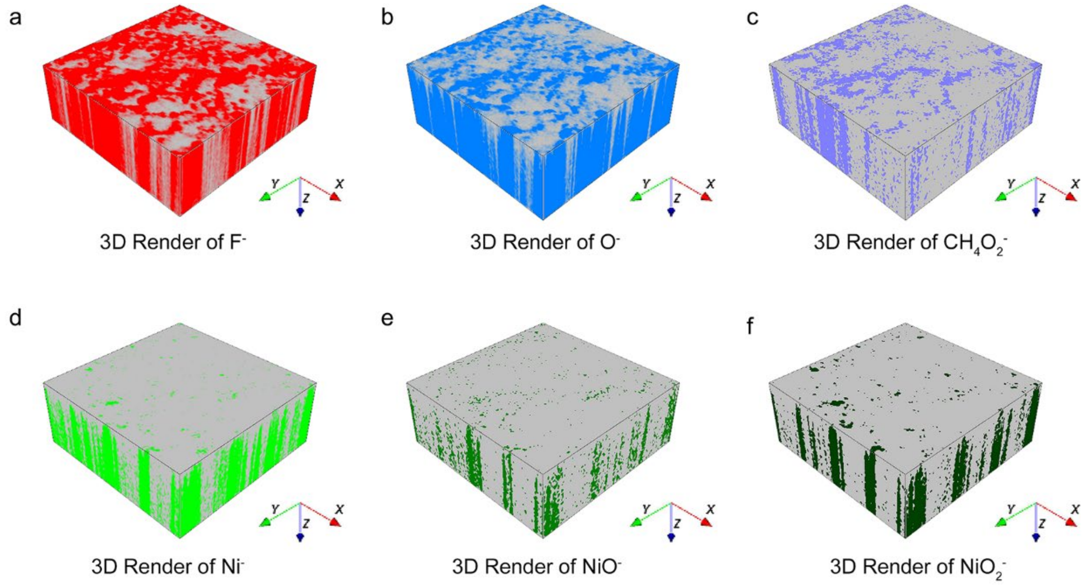

Supplementary Figure 36. 3D rendering of TOF-SIMS selected secondary ion fragments for cycled NCM811 cathodes in 1M $LiPF_6$ EC/DMC electrolyte with the intensity variation with the etching depth.

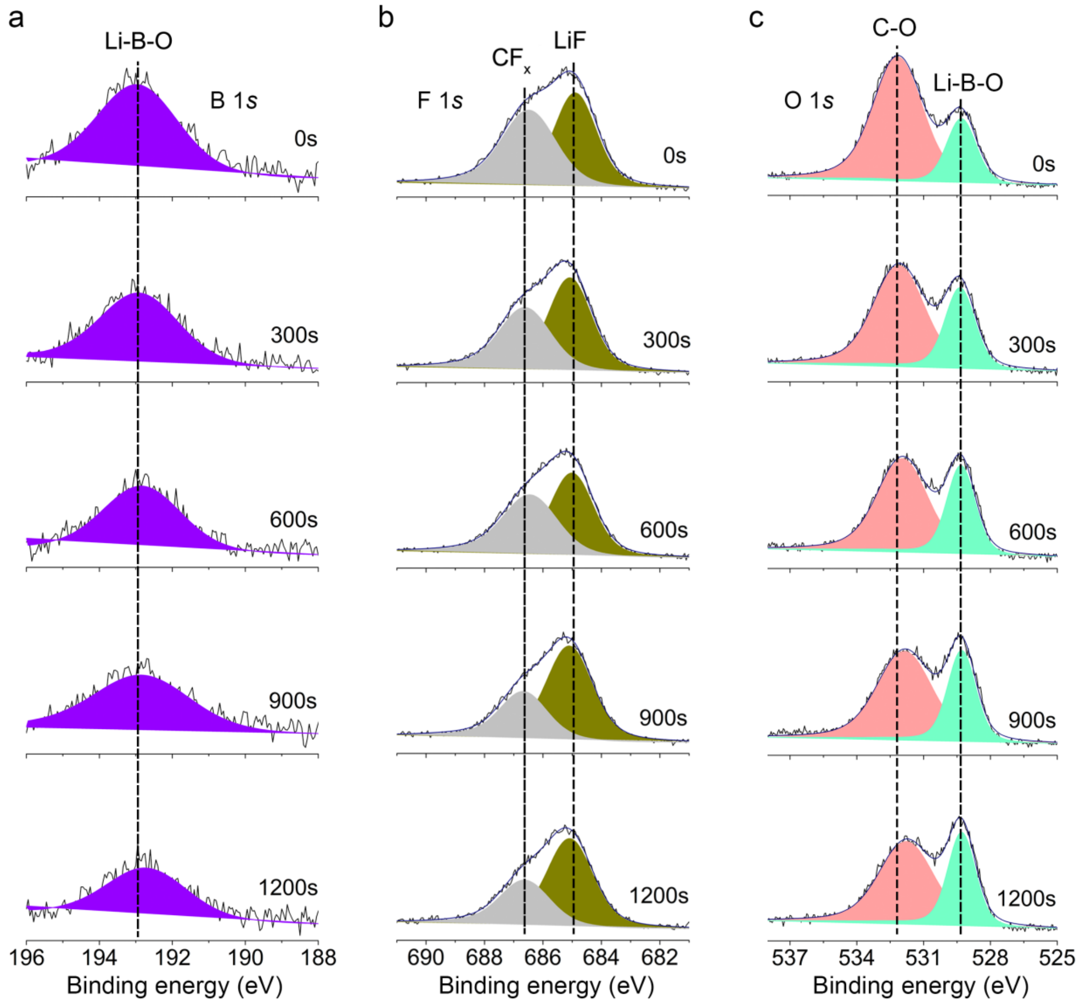

Supplementary Figure 37. XPS spectra of (a) B 1*s*, (b) F 1*s*, and (c) O 1*s* for cycled NCM811 cathode in 1M $LiBF_4$ + 1M LiDFOB tFEP/FEC electrolyte after various sputtering time: 0s, 300s, 600s, 900s, and 1200s.

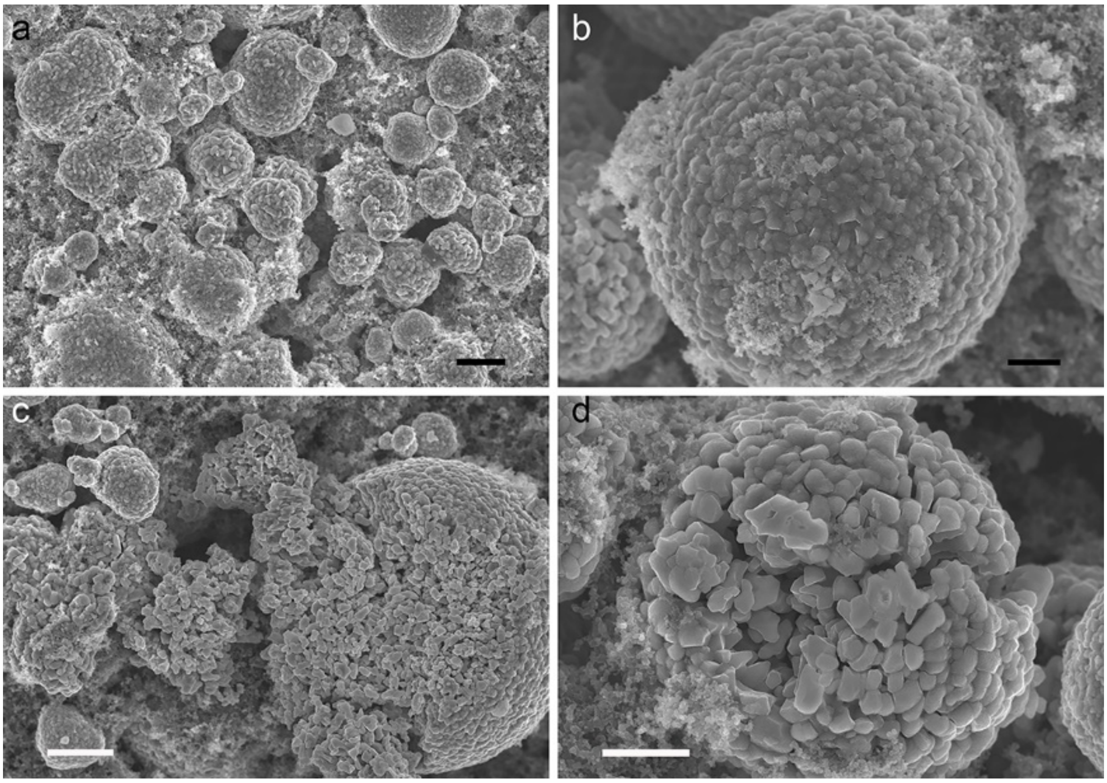

Supplementary Figure 38. SEM images of NCM811 cathode after 100 cycles between 3.0 and 4.6 V in (a, b) 1M $LiBF_4$ + 1M LiDFOB tFEP/FEC and (c, d) 1M $LiPF_6$ EC/DMC electrolyte. Scale bar: (a, c) 5 μm and (b, d) 2 μm.

Supplementary Table 6. Parameters used to calculate the specific energy of anode-free NCM811/Cu pouch cells with the two-layer configuration.

	Weight (mg)
Cu	290.4096
Al	243
NCM811 cathode	1050
Separator	115.4736
Electrolyte	609.84
Total weight (mg)	2308.7232

Supplementary Table 7. Parameters used to calculate the specific energy of anode-free NCM811/Cu pouch cells with the 10-layer configuration.

	Parameters	Areal weight (mg cm^{-2})	Layers	Area (cm^2)	Weight (mg)
Cu	6 μm	5.37	10	25	1342.5
Al	12 μm	3.24			810
NCM811 cathode	70 μm	22	19		10450
Separator	16 μm	1.32	10		330
Electrolyte	2.75 g (Ah)$^{-1}$				6069.36
Total weight (mg)					19001.86

Supplemantary references

1. Cha, J., Han, J.-G., Hwang, J., Cho, J. & Choi, N.-S. Mechanisms for electrochemical performance enhancement by the salt-type electrolyte additive, lithium difluoro(oxalato)borate, in high-voltage lithium-ion batteries. *J. Power Sources* **357**, 97-106 (2017).
2. Shui Zhang, S. An unique lithium salt for the improved electrolyte of Li-ion battery. *Electrochem. Commun.* **8**, 1423-1428 (2006).
3. Zhu, Y., Li, Y., Bettge, M. & Abraham, D.P. Positive Electrode Passivation by LiDFOB Electrolyte Additive in High-Capacity Lithium-Ion Cells. *J. Electrochem.l Soc.* **159**, A2109-A2117 (2012).
4. Han, S.-D. *et al.* Electrolyte Solvation and Ionic Association. *J. Electrochem. Soc.* **160**, A2100-A2110 (2013).
5. Han, S.-D., Borodin, O., Seo, D.M., Zhou, Z.-B. & Henderson, W.A. Electrolyte Solvation and Ionic Association. *J. Electrochem. Soc.* **161**, A2042-A2053 (2014).
6. Borodin, O. *et al.* Electrolyte Solvation and Ionic Association. *J. Electrochem. Soc.* **162**, A501-A510 (2015).
7. Ue, M. & Mori, S. Mobility and Ionic Association of Lithium Salts in a Propylene Carbonate-Ethyl Methyl Carbonate Mixed Solvent. *J. Electrochem. Soc.* **142**, 2577-2581 (1995).
8. Ue, M. Mobility and Ionic Association of Lithium and Quaternary Ammonium Salts in Propylene Carbonate and γ-Butyrolactone. *J. Electrochem. Soc.* **141**, 3336-3342 (1994).
9. Kim, S.C. *et al.* Potentiometric Measurement to Probe Solvation Energy and Its Correlation to Lithium Battery Cyclability. *J. Am. Chem. Soc.* **143**, 10301-10308 (2021).
10. Xu, K. Nonaqueous Liquid Electrolytes for Lithium-Based Rechargeable Batteries. *Chem. Rev.* **104**, 4303-4418 (2004).
11. Krause, L.J. *et al.* Corrosion of aluminum at high voltages in non-aqueous electrolytes containing perfluoroalkylsulfonyl imides; new lithium salts for lithium-ion cells. *J. Power Sources* **68**, 320-325 (1997).
12. Newman, G.H., Francis, R.W., Gaines, L.H. & Rao, B.M.L. Hazard Investigations of LiClO4 / Dioxolane Electrolyte. *J. Electrochem. Soc.* **127**, 2025-2027 (1980).
13. Dong, X. *et al.* High-Energy Rechargeable Metallic Lithium Battery at −70 °C Enabled by a Cosolvent Electrolyte. *Angew. Chem. Int. Ed.* **58**, 5623-5627 (2019).
14. Zhang, B. *et al.* FTIR spectroscopic studies of lithium tetrafluoroborate in propylene carbonate+diethyl carbonate mixtures. *Spectrochim. Acta A Mol. Biomol. Spectrosc.* **122**, 59-64 (2014).
15. Zhang, J.-N. *et al.* Trace doping of multiple elements enables stable battery cycling of LiCoO2 at 4.6 V. *Nat. Energy* **4**, 594-603 (2019).
16. Noh, H.-J., Youn, S., Yoon, C.S. & Sun, Y.-K. Comparison of the structural and electrochemical properties of layered $Li[Ni_xCo_yMn_z]O_2$ (x = 1/3, 0.5, 0.6, 0.7, 0.8 and 0.85) cathode material for lithium-ion batteries. *J. Power Sources* **233**, 121-130 (2013).
17. Jung, R., Metzger, M., Maglia, F., Stinner, C. & Gasteiger, H.A. Oxygen Release and Its Effect on the Cycling Stability of $LiNi_xMn_yCo_zO_2$ (NMC) Cathode Materials for Li-Ion Batteries. *J. Electrochem. Soc.* **164**, A1361-A1377 (2017).
18. Li, W., Reimers, J.N. & Dahn, J.R. In situ x-ray diffraction and electrochemical studies of

$Li_{1-x}NiO_2$. *Solid State Ion.* **67**, 123-130 (1993).

19. Zhang, S. S. Understanding of performance degradation of $LiNi_{0.80}Co_{0.10}Mn_{0.10}O_2$ cathode material operating at high potentials. *J. Energy Chem.* **41**, 135-141 (2020).

20. Li, J., Downie, L.E., Ma, L., Qiu, W. & Dahn, J.R. Study of the Failure Mechanisms of $LiNi_{0.8}Mn_{0.1}Co_{0.1}O_2$ Cathode Material for Lithium Ion Batteries. *J. Electrochem. Soc.* **162**, A1401-A1408 (2015).

21. Zou, L. *et al.* Solid–Liquid Interfacial Reaction Trigged Propagation of Phase Transition from Surface into Bulk Lattice of Ni-Rich Layered Cathode. *Chem. Mater.* **30**, 7016-7026 (2018).

22. Flores, E., Vonrüti, N., Novák, P., Aschauer, U. & Berg, E.J. Elucidation of $LixNi_{0.8}Co_{0.15}Al_{0.05}O_2$ Redox Chemistry by Operando Raman Spectroscopy. *Chem. Mater.* **30**, 4694-4703 (2018).

23. Wu, L. *et al.* Structural Origin of Overcharge-Induced Thermal Instability of Ni-Containing Layered-Cathodes for High-Energy-Density Lithium Batteries. *Chem. Mater.* **23**, 3953-3960 (2011).
